# Public Health Interventions and Overdose-Related Outcomes Among Persons With Opioid Use Disorder

**DOI:** 10.1001/jamanetworkopen.2024.4617

**Published:** 2024-04-03

**Authors:** Nisha Nataraj, S. Michaela Rikard, Kun Zhang, Xinyi Jiang, Gery P. Guy, Ketra Rice, Christine L. Mattson, R. Matthew Gladden, Desiree M. Mustaquim, Zachary N. Illg, Puja Seth, Rita K. Noonan, Jan L. Losby

**Affiliations:** 1 Division of Overdose Prevention, National Center for Injury Prevention and Control, US Centers for Disease Control and Prevention, Atlanta, Georgia; 2 Division of Injury Prevention, National Center for Injury Prevention and Control, US Centers for Disease Control and Prevention, Atlanta, Georgia; 3 Department of Emergency Medicine, Emory University School of Medicine, Atlanta, Georgia

## Abstract

**Importance:**

Given the high number of opioid overdose deaths in the US and the complex epidemiology of opioid use disorder (OUD), systems models can serve as a tool to identify opportunities for public health interventions.

**Objective:**

To estimate the projected 3-year association between public health interventions and opioid overdose-related outcomes among persons with OUD.

**Design, Setting, and Participants:**

This decision analytical model used a simulation model of the estimated US population aged 12 years and older with OUD that was developed and analyzed between January 2019 and December 2023. The model was parameterized and calibrated using 2019 to 2020 data and used to estimate the relative change in outcomes associated with simulated public health interventions implemented between 2021 and 2023.

**Main Outcomes and Measures:**

Projected OUD and medications for OUD (MOUD) prevalence in 2023 and number of nonfatal and fatal opioid-involved overdoses among persons with OUD between 2021 and 2023.

**Results:**

In a baseline scenario assuming parameters calibrated using 2019 to 2020 data remained constant, the model projected more than 16 million persons with OUD not receiving MOUD treatment and nearly 1.7 million persons receiving MOUD treatment in 2023. Additionally, the model projected over 5 million nonfatal and over 145 000 fatal opioid-involved overdoses among persons with OUD between 2021 and 2023. When simulating combinations of interventions that involved reducing overdose rates by 50%, the model projected decreases of up to 35.2% in nonfatal and 36.6% in fatal opioid-involved overdoses among persons with OUD. Interventions specific to persons with OUD not currently receiving MOUD treatment demonstrated the greatest reduction in numbers of nonfatal and fatal overdoses. Combinations of interventions that increased MOUD initiation and decreased OUD recurrence were projected to reduce OUD prevalence by up to 23.4%, increase MOUD prevalence by up to 137.1%, and reduce nonfatal and fatal opioid-involved overdoses among persons with OUD by 6.7% and 3.5%, respectively.

**Conclusions and Relevance:**

In this decision analytical model study of persons with OUD, findings suggested that expansion of evidence-based interventions that directly reduce the risk of overdose fatality among persons with OUD, such as through harm reduction efforts, could engender the highest reductions in fatal overdoses in the short-term. Interventions aimed at increasing MOUD initiation and retention of persons in treatment projected considerable improvement in MOUD and OUD prevalence but could require a longer time horizon for substantial reductions in opioid-involved overdoses.

## Key Points

**Question:**

What is the projected association between opioid overdose-related outcomes among US persons with opioid use disorder (OUD) and public health interventions to increase treatment prevalence and decrease overdose rates?

**Findings:**

In this decision analytical model, simulated public health interventions, combined, among more than 16 million projected persons with OUD not receiving medication for OUD (MOUD) and nearly 1.7 million projected persons receiving MOUD were estimated to decrease OUD prevalence by 23%, increase the prevalence of MOUD treatment by 137%, and decrease nonfatal and fatal opioid-involved overdoses by 35% and 37%, respectively, between 2021 and 2023.

**Meaning:**

These findings suggest that a multifaceted public health approach, including increased access, linkage to, and retention in treatment and overdose education and harm reduction efforts to reduce overdose rates, is important to improve outcomes among persons with OUD.

## Teaser

This decision analytical model estimates the projected 3-year association between public health interventions and opioid overdose-related outcomes among persons with opioid use disorder (OUD).

## Introduction

Opioid-involved overdose deaths remain at high levels in the US, driven primarily by synthetic opioids such as illegally made fentanyl (IMF).^1,2^ In 2021, 80 816 opioid-involved overdose deaths occurred,^1^ and an estimated 5.6 million persons aged 12 years and older had an opioid use disorder (OUD) in the US, based on the *Diagnostic and Statistical Manual of Mental Disorders* (Fifth Edition) criteria for heroin or prescription pain reliever use disorder in the National Survey on Drug Use and Health (NSDUH).^3^ Medications for OUD (MOUD)—buprenorphine, naltrexone, and methadone—approved for OUD treatment by the Food and Drug Administration are highly effective treatments to support recovery from OUD and reduce risk of opioid overdose.^4,5,6,7^ Expanding access to MOUD treatment is a cornerstone of the response to the opioid overdose crisis.^8,9^

Given the complexity of OUD and the epidemic of opioid-involved overdose deaths, systems models have utility as a tool to understand the potential impact of and opportunities for evidence-based interventions to prevent overdose.^10^ Several systems models examining opioid use, misuse, OUD, and resulting overdose have been recently developed.^11,12,13,14,15,16,17^ While these models differ in scope, they highlight the need to combine interventions to achieve considerable reductions in opioid-involved overdose deaths. However, only a few state-specific models exist that focus on the population with OUD,^12^ and previous models incorporate limited information on MOUD treatment duration. We developed a national-level simulation model of persons with OUD, Modeling OUD Dynamics Informing Public Health Interventions (MODIPHI), to estimate the relative change in opioid overdose-related outcomes that can be achieved by scaling public health interventions among persons with OUD in the short-term. This model additionally leverages new treatment and fatal overdose datasets to improve our understanding of population dynamics associated with recovery from OUD, including the risk of OUD recurrence and nonfatal and fatal overdose risk by explicitly modeling different durations of MOUD treatment. Findings from this model can provide insights about the potential outcomes associated with increasing population reach of or investments in public health interventions among people with OUD in the short term.

## Methods

We developed and calibrated MODIPHI, a national-level system dynamics simulation model^18^ of the estimated US population aged 12 years and older with OUD using data from 2019 to 2020. The calibrated model was used to estimate the association between overdose prevention interventions simulated between 2021 and 2023, the prevalence of OUD and MOUD, and the number of nonfatal and fatal opioid-involved overdoses among persons with OUD. This study was reviewed by the US Centers for Disease Control and Prevention (CDC) and conducted consistent with applicable federal law and CDC policy in accordance with 45 CFR §46. Institutional review board approval was not sought and the need for informed consent did not apply because deidentified, retrospective, aggregate data were used in this study. This study followed the Consolidated Health Economic Evaluation Reporting Standards (CHEERS) reporting guideline.^19^

### Disease Model of OUD

**Figure 1.**
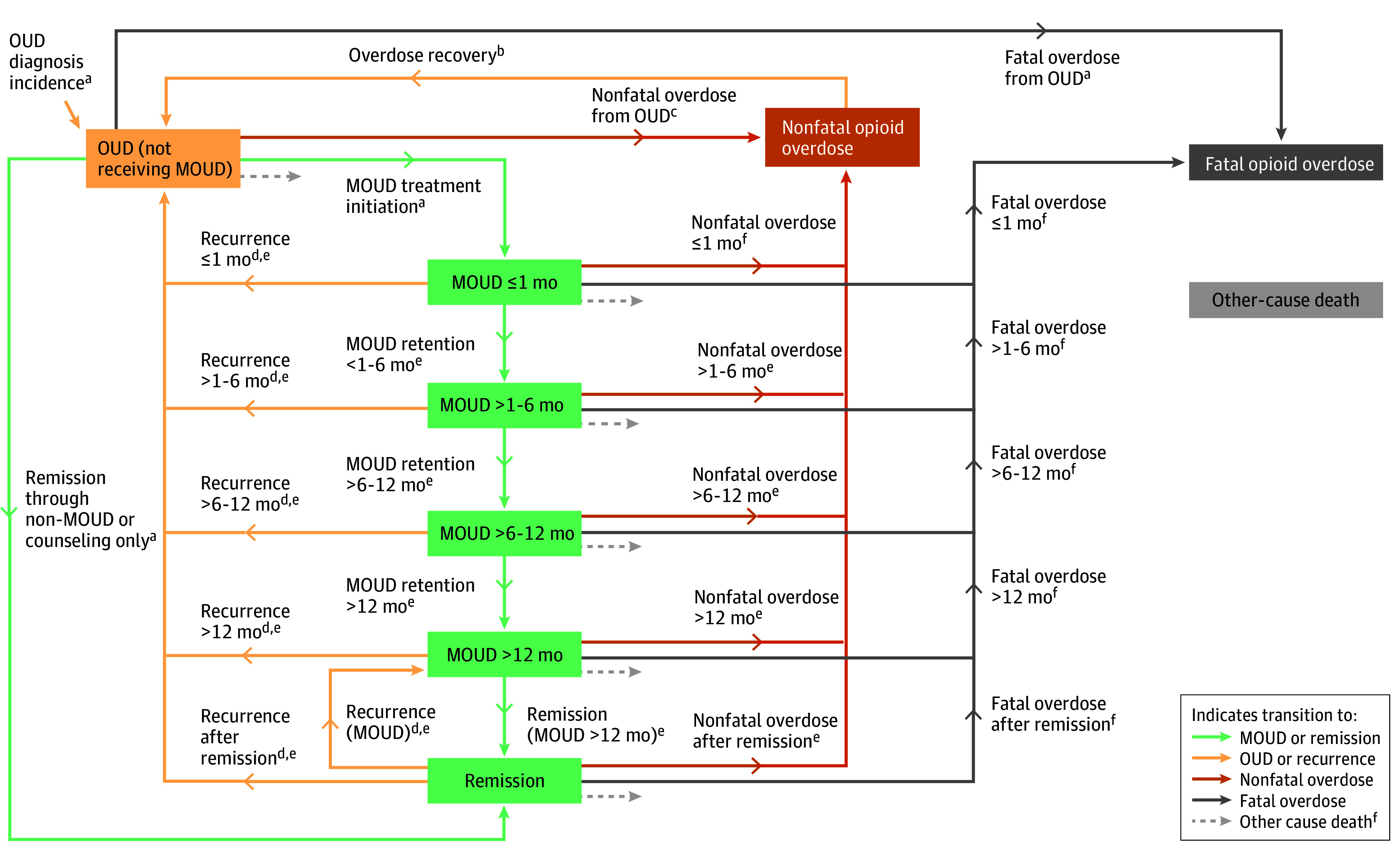
Schematic of Natural History Model of Opioid Use Disorder (OUD) and Medications for Opioid Use Disorder (MOUD) ^a^Parameters were calibrated. ^b^Transition is modeled with a time delay of 1 day. ^c^Parameters derived from MarketScan Commercial and Multi-State Medicaid Databases. ^d^Recurrence of OUD as defined by recent opioid use and discontinuation of MOUD. ^e^Parameters derived from MOUD study^20^ data. ^f^Parameters derived from scientific literature.

The disease model comprised 9 model states: (1) persons with OUD (not receiving MOUD treatment), (2) persons with OUD receiving MOUD treatment with durations of 1 month or less, (3) more than 1 month to 6 months or less, (4) more than 6 months to 12 months or less, and (5) more than 12 months; (6) persons in sustained remission from OUD (defined here as receiving MOUD or counseling-only treatment for at least 360 days and no reported opioid use in the past 90 days); (7) nonfatal opioid-involved overdose; (8) fatal opioid-involved overdose; and (9) death from other causes (Figure 1). Persons in the OUD model state could initiate MOUD, enter remission without MOUD, or experience a nonfatal or fatal overdose or death from other causes. We assumed all persons who experienced a nonfatal overdose subsequently transitioned to the OUD model state after a time delay of 1 day, regardless of their prior model state, but could once again initiate MOUD treatment. Persons in any MOUD state or remission could experience nonfatal or fatal overdoses, a death from other causes, or recurrence of OUD (defined as discontinuation of MOUD combined with past 90-day opioid use). Annually, persons newly diagnosed with OUD were added to the OUD model state. eAppendix 1 in Supplement 1 provide details of model states and the study population.

### Data and Parameter Estimation

eAppendix 2 in Supplement 1 provides details of all model parameters including descriptions, values, and data sources (eTable 1 in Supplement 1). Data from CDC’s MOUD study were used to parameterize transitions from MOUD model states including recurrence, nonfatal overdose, and remission (eAppendix 3 in Supplement 1).^20^ The CDC MOUD study was an 18-month longitudinal cohort study, evaluating participants with OUD (aged ≥18 years) receiving outpatient treatment for OUD, which included methadone, buprenorphine, and naltrexone, alone or in combination with behavioral therapy. Participants completed baseline questionnaires upon study enrollment, and again at approximately 3, 6, 12, and 18 months following enrollment. We parameterized model transitions from the questionnaire responses, which included self-reported individual-level data about length of time in treatment, completion or cessation of treatment, past 30-day and 90-day opioid use, and past 90-day opioid overdose.

We used MarketScan Commercial and Multi-State Medicaid Databases, accessed through the MarketScan Treatment Pathways platform, to obtain nonfatal overdose rates among persons diagnosed with OUD not receiving MOUD (eAppendix 4, eTable 2, and eTable 3 in Supplement 1). Nonfatal overdose rates among persons receiving MOUD for less than 1 month, and rates of fatal overdose and death from other causes among persons receiving MOUD and those in remission were obtained from scientific literature since they could not be determined from the MOUD Study^20^ or other data sources (eAppendix 5 in Supplement 1). The model was initialized on January 1, 2019 (eTable 4 in Supplement 1), with prevalence estimates of OUD obtained from the 2018 NSDUH Annual Report,^21^ adjusted for national prevalence via the previously reported benchmark multiplier method^22^ (eAppendix 1 and 6 in Supplement 1).

### Baseline Model Development, Calibration, and Validation

The system dynamics model was developed in AnyLogic version 8.7.10 (The AnyLogic Company)^23^ and calibrated 7 parameters (eTable 1 in Supplement 1) to 3 targets—fatal overdoses, OUD prevalence, and MOUD prevalence (eTable 5 in Supplement 1)—using the OptQuest optimizer engine^24^ within AnyLogic. To calibrate fatal overdoses, we used data from CDC’s State Unintentional Drug Overdose Reporting System (SUDORS) to estimate the likelihood of OUD among persons who experienced a fatal opioid-involved overdose by identifying decedents who had evidence of current or past prescription opioid misuse, heroin use, or treatment for substance use disorder.^25^ SUDORS collects information on drug overdose deaths of unintentional and undetermined intent using information from death certificates and medical examiner and coroner reports, including postmortem toxicology results.^25^ Together with opioid-involved overdose mortality data from the National Vital Statistics System, we estimated that the national fatal opioid-involved overdoses among persons with OUD in 2019 and 2020 were 24 796 and 34 131, respectively, which we used as a calibration target (eAppendix 7, eTable 6, and eTable 7 in Supplement 1). We also calibrated the model to adjusted 2019 to 2020 NSDUH estimates^26,27^ of OUD prevalence (eAppendix 8 in Supplement 1). We adjusted prevalence data reported in NSDUH since estimates of OUD from general population surveys have been shown to underestimate prevalence by up to 3- to 5-fold.^22,28^ Additional calibration targets included MOUD prevalence (eAppendix 9 in Supplement 1) with calibration results reported in eAppendix 10 in Supplement 1 (eFigure 1 and eTable 8 in Supplement 1). The projected number of nonfatal overdoses was validated with a recent estimate in the literature^29^ (eAppendix 11 in Supplement 1).

### Simulating Public Health Interventions

Evidence from previous models highlights the importance of combining interventions to achieve reductions in opioid overdose-related outcomes.^12,15^ As in previous studies, we simulated hypothetical improvements^13,17^ directly in the rates of transitions in the OUD disease model (eg, increase in initiation of treatment with MOUD, reduction in recurrence of OUD [ie, discontinuation of MOUD combined with past 90-day opioid use], and reduction of nonfatal and fatal opioid overdose rates) resulting from combinations of unspecified public health interventions. We chose this approach because limited data exist to precisely estimate the impact of evidence-based interventions when scaled at a national level. By focusing on the intended goals achieved through an unspecified intervention, we can better identify which transition pathways and intermediate outcomes are most critical to improving opioid overdose-related outcomes. In this study, we modeled 4 possible intervention scenarios aiming to yield the following: scenario A increased MOUD initiation and decreased OUD recurrence among persons receiving MOUD for 6 months or less (defined as early-stage) and persons receiving MOUD for more than 6 months (defined as late-stage); scenario B decreased fatal overdose rates and decreased recurrence of OUD; scenario C decreased nonfatal overdose rates and decreased recurrence of OUD; and scenario D increased MOUD initiation and decreased fatal overdose rates.

To simulate the change in specific model transitions from combined interventions, the corresponding parameters were increased or decreased by a percentage of the baseline parameter value. Most parameters were decreased by 10% to 50% of the original parameter value, except for the MOUD initiation rate, which was increased by 50% to 200%. We chose this larger range for MOUD initiation given substantial variations and reduction in MOUD prevalence observed in NSDUH between 2019 and 2020 (18.1% in 2019 vs 11.2% in 2020 of persons aged 12 years or older with a past year OUD).^26,27^ These increases or decreases in parameter values were implemented stepwise between 2021 and 2023, representing gradual implementation of the intervention. For example, a 50% overall decrease in a parameter across the 3-year simulation time horizon was simulated by implementing a 16.7% stepwise decrease in the original parameter value at the beginning of each year to achieve a 50% decrease in the final year.

### Data Analysis

We reported model estimates for outcomes of interest (nonfatal and fatal opioid overdoses, OUD prevalence, and MOUD prevalence) as a percentage change relative to projected outcomes in the baseline model scenario at the end of 2023. One-way sensitivity analyses were also conducted on model parameters as well as OUD and fatal overdose calibration targets within AnyLogic.

## Results

The baseline model projected outcomes through 2023, assuming parameter values calibrated with 2019 to 2020 historical data remained constant between 2021 and 2023 (eTable 8 in Supplement 1). The baseline model projected 16 072 360 persons with OUD not receiving MOUD and 1 677 988 persons receiving MOUD at the end of 2023 and 5 102 289 nonfatal and 145 237 fatal opioid-involved overdoses among persons with OUD between 2021 and 2023. Additional outcomes are reported in eAppendix 12, eTable 9, eTable 10, eTable 11, and eFigure 2 in Supplement 1. Results from the sensitivity analyses demonstrating robustness of the main findings are reported in eAppendix 13 (eFigure 3, eFigure 4, eFigure 5, eFigure 6, eFigure 7, eFigure 8), eAppendix 14 (eTable 12, and eFigure 9) and eAppendix 15 (eTable 13) in Supplement 1.

### Scenario A: Increased MOUD Initiation and Decreased OUD Recurrence

**Figure 2.**
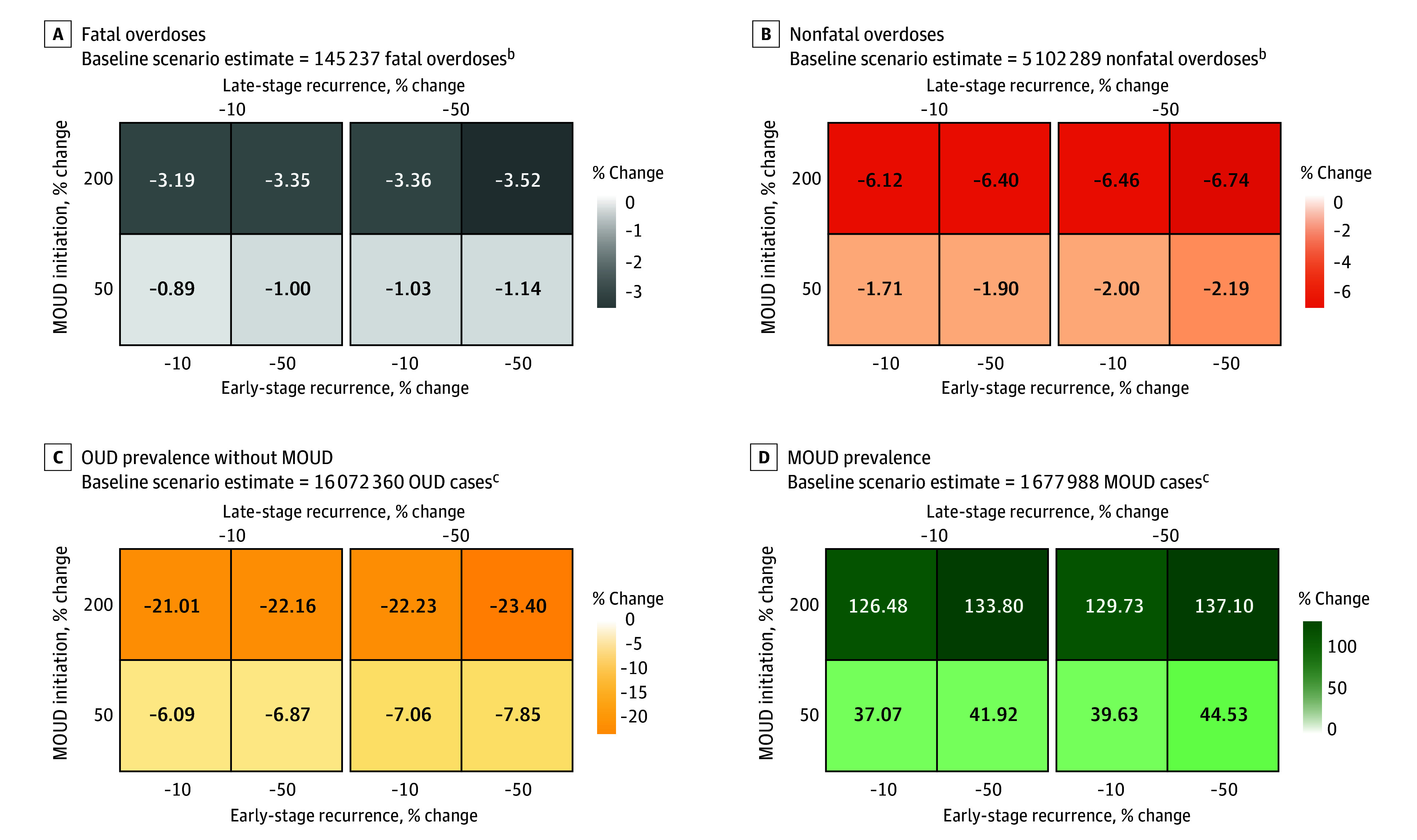
Percentage Change in Projected Model Outcomes Relative to the Baseline Scenario for Fatal Overdoses, Nonfatal Overdoses, Opioid Use Disorder (OUD) Prevalence Without Medications for Opioid Use Disorder (MOUD), and MOUD Prevalence Under Model Scenario A^a^ ^a^Increased MOUD initiation and decreased early- and late-stage OUD recurrence. ^b^Model estimates are cumulative over the time horizon of simulated public health interventions (January 1, 2021, to December 31, 2023). ^c^Model estimates indicate prevalence at the end of the simulation (December 31, 2023).

Increasing the rate of MOUD initiation among persons with OUD by 200% (from 10.2% to 30.6%), while simultaneously decreasing early- and late-stage OUD recurrence rates by 50%, was estimated to reduce OUD prevalence (3 761 723 persons [−23.4%]), increase MOUD prevalence (2 300 594 persons [137.1%]), reduce nonfatal overdoses (343 947 nonfatal overdoses [−6.7%]), and reduce fatal overdoses (5106 fatal overdoses [−3.5%]) (Figure 2). Compared with reducing late-stage OUD recurrence, reducing OUD recurrence rates among early-stage MOUD treatment was associated with a slightly larger change in MOUD prevalence. Given a 200% increase in MOUD initiation rate, decreasing early-stage OUD recurrence rate by 50% resulted in higher MOUD prevalence compared with the same decrease in late-stage OUD recurrence rate (133.8% vs 129.7% increase in MOUD prevalence).

### Scenario B: Decreased Fatal Overdoses and Decreased OUD Recurrence

**Figure 3.**
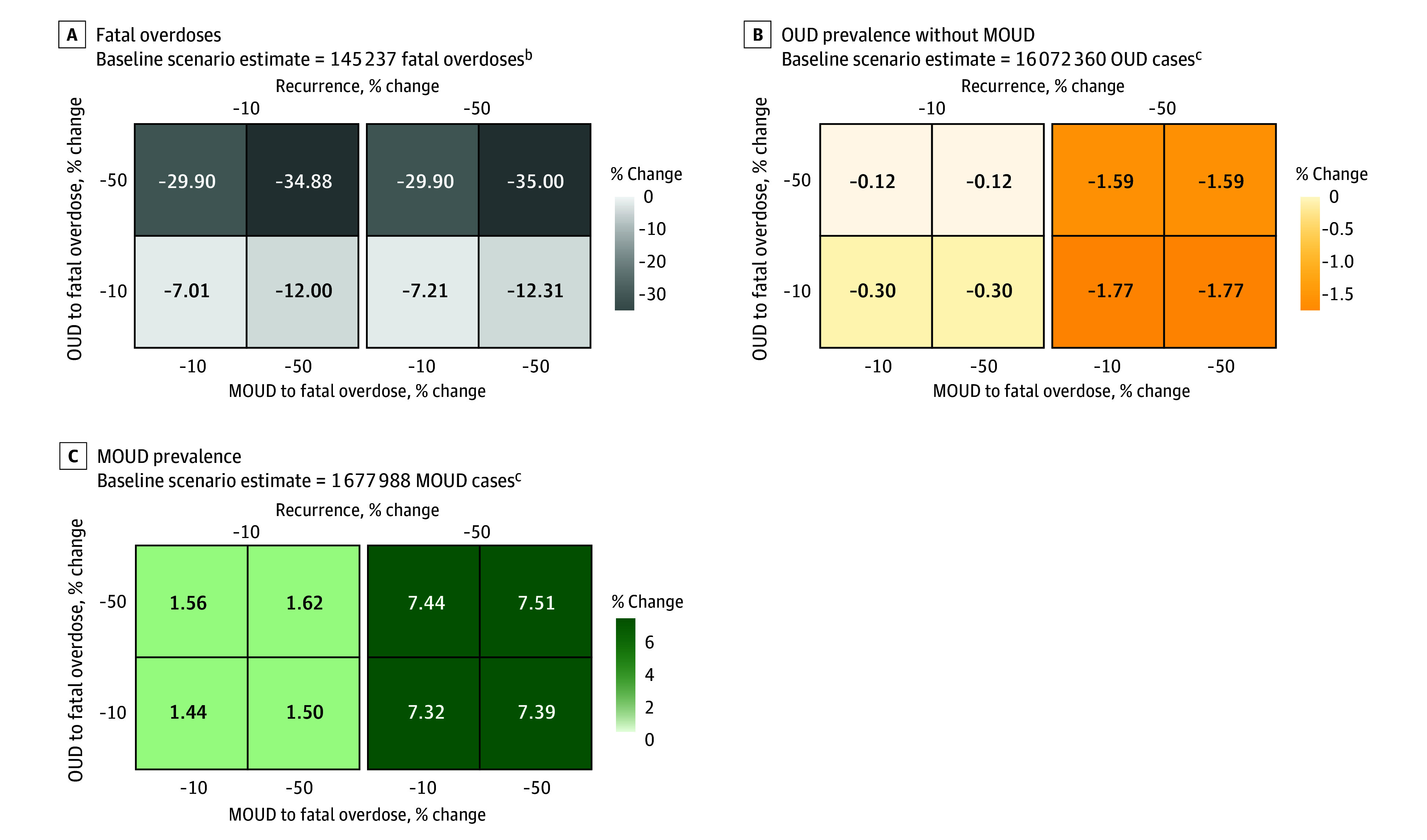
Percentage Change in Projected Model Outcomes Relative to the Baseline Scenario for Fatal Overdoses, Opioid Use Disorder (OUD) Prevalence Without Medications for Opioid Use Disorder (MOUD), and MOUD Prevalence Under Model Scenario B^a^ ^a^Decreased fatal overdoses and decreased OUD recurrence. ^b^Model estimates are cumulative over the time horizon of simulated public health interventions (January 1, 2021, to December 31, 2023). ^c^Model estimates indicate prevalence at the end of the simulation (December 31, 2023).

Decreasing fatal overdose rates by 50% among populations with OUD and those receiving MOUD, while simultaneously decreasing OUD recurrence rates by 50%, was estimated to reduce OUD prevalence (255 171 persons [−1.6%]), increase MOUD prevalence (126 001 persons [7.5%]), and reduce fatal overdoses (50 839 fatal overdoses [−35.0%]) (Figure 3). Decreasing the fatal overdose rate by 50% among persons with OUD not receiving MOUD resulted in an additional 17.9% decrease in fatal opioid overdoses compared with interventions that similarly yielded decreased fatal overdose rates among persons receiving MOUD (from −12.0% to −29.9%). Decreasing the fatal overdose rate among persons with OUD not receiving MOUD demonstrated the largest reduction in fatal opioid overdoses due to the high proportion of this population. Decreasing OUD recurrence rates had minimal association with fatal opioid overdoses but was estimated to decrease OUD prevalence (from −0.1% to −1.6%) and increase MOUD prevalence (from 1.6% to 7.5%).

### Scenario C: Decreased Nonfatal Overdoses and Decreased OUD Recurrence

**Figure 4.**
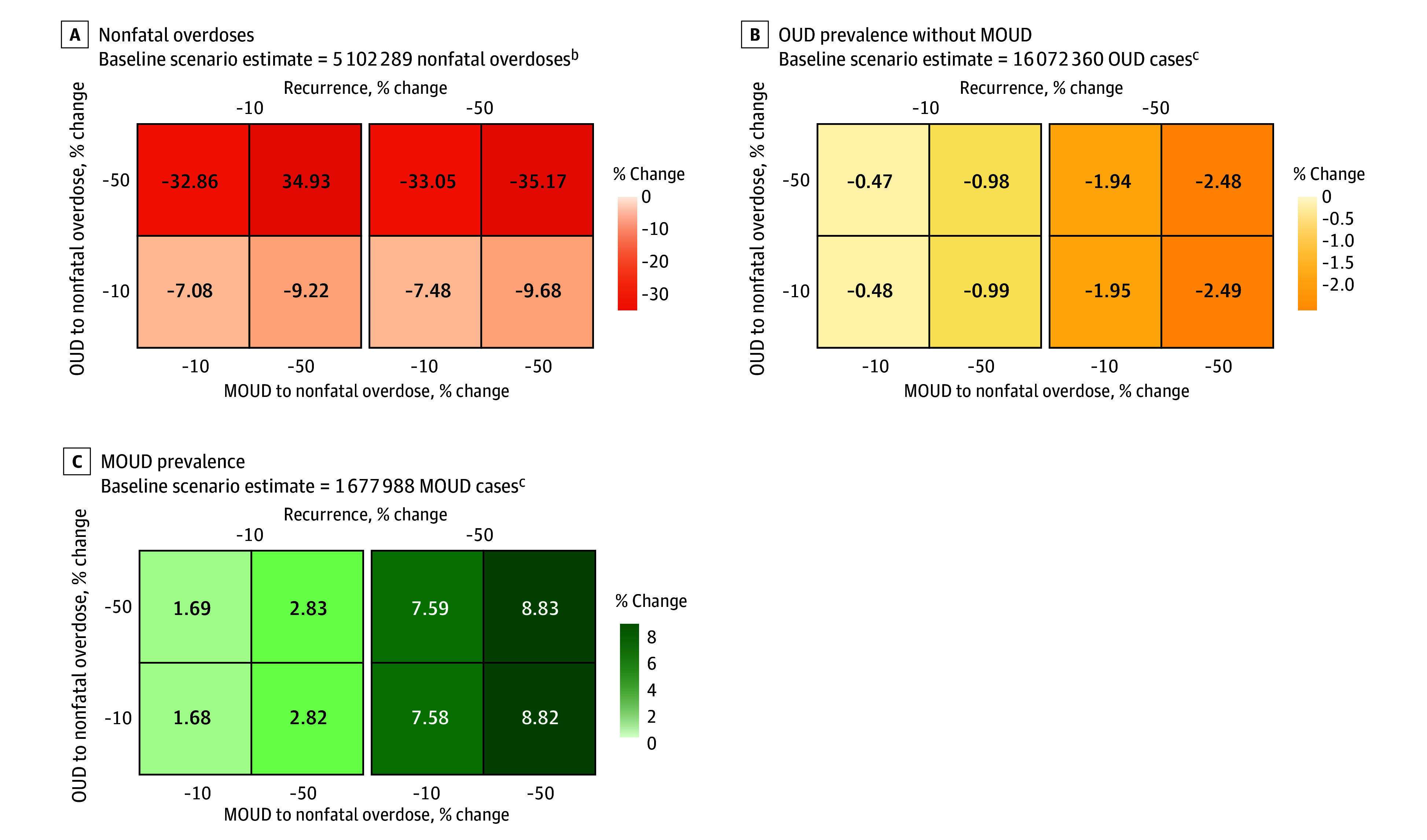
Percentage Change in Projected Model Outcomes Relative to the Baseline Scenario for Nonfatal Overdoses, Opioid Use Disorder (OUD) Prevalence Without Medications for Opioid Use Disorder (MOUD), and MOUD Prevalence Under Model Scenario C^a^ ^a^Decreased nonfatal overdoses and decreased OUD recurrence. ^b^Model estimates are cumulative over the time horizon of simulated public health interventions (January 1, 2021, to December 31, 2023). ^c^Model estimates indicate prevalence at the end of the simulation (December 31, 2023).

Decreasing nonfatal overdose rates by 50% among populations with OUD and those receiving MOUD, while simultaneously decreasing OUD recurrence rates by 50%, was estimated to reduce OUD prevalence (399 218 persons [−2.5%]), increase MOUD prevalence (148 110 persons [8.8%]), and reduce nonfatal overdoses (1 794 708 nonfatal overdoses [−35.2%]) (Figure 4). Similar to interventions that yielded decreased fatal overdose rates (scenario B), decreasing the nonfatal overdose rate among populations with OUD not receiving MOUD demonstrated the largest reduction in nonfatal overdoses due to the higher proportion of persons in this category. Decreasing nonfatal overdose rates by 50% among persons with OUD not receiving MOUD resulted in an additional 23.6% decrease in nonfatal overdoses compared with a similar intervention among persons receiving MOUD (from −9.2% to −32.9%). Decreasing OUD recurrence rates had minimal association with nonfatal overdoses but was estimated to decrease OUD prevalence (from −1.0% to −2.5%) and increase MOUD prevalence (from 2.8% to 8.8%).

### Scenario D: Decreased Fatal Overdoses and Increased MOUD Initiation

**Figure 5.**
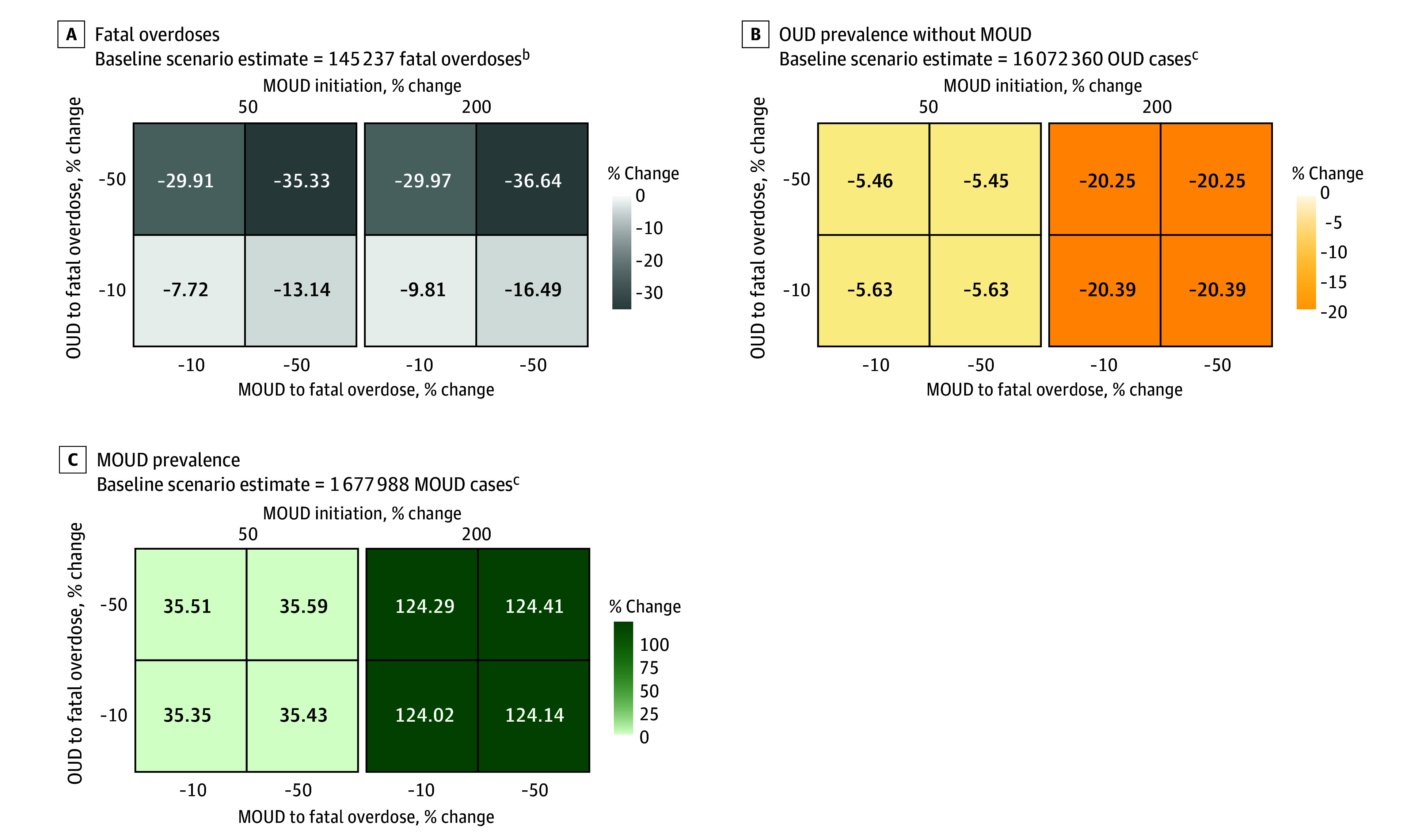
Percentage Change in Projected Model Outcomes Relative to the Baseline Scenario for Fatal Overdoses, Opioid Use Disorder (OUD) Prevalence Without Medications for Opioid Use Disorder (MOUD), and MOUD Prevalence Under Model Scenario D^a^ ^a^Decreased fatal overdoses and increased MOUD initiation. ^b^Model estimates are cumulative over the time horizon of simulated public health interventions (January 1, 2021, to December 31, 2023). ^c^Model estimates indicate prevalence at the end of the simulation (December 31, 2023).

Increasing the rate of MOUD initiation among persons with OUD by 200% (from 10.2% to 30.6%), while simultaneously decreasing fatal overdose rates among populations with OUD and those receiving MOUD, was estimated to reduce OUD prevalence (3 254 168 persons [−20.3%]), increase MOUD prevalence (2 087 601 persons [124.4%]), and reduce fatal overdoses (53 217 fatal overdoses [−36.6%]) (Figure 5). Decreasing the fatal overdose rate by 50% among persons with OUD not receiving MOUD resulted in an additional 16.8% decrease in fatal overdoses compared with a similar intervention among persons receiving MOUD (from −13.1% to −29.9%). Decreasing fatal overdose rates had minimal association with OUD and MOUD prevalence but increasing MOUD initiation rate from 50% to 200% substantially reduced OUD prevalence (from −5.6% to −20.4%) and increased MOUD prevalence (from 35.6% to 124.4%).

## Discussion

This national-level system dynamics model of OUD, MODIPHI, representing OUD treatment and recovery population dynamics, estimated the relative change in overdose-related outcomes from scaling public health interventions during a 3-year period. To our knowledge, this model is the first to explicitly incorporate MOUD treatment duration by leveraging treatment data and fatal overdose data from the CDC MOUD Study^20^ and SUDORS. Furthermore, this is one of few existing simulation models that projects nonfatal overdoses, an important metric in mitigating the overdose epidemic. The inclusion of several overdose-related outcomes provides insight about the potential impacts of multiple public health interventions and underlying population dynamics driving recovery from OUD.

Evidence-based interventions intended to reduce risk of fatal opioid overdose, such as through harm reduction efforts (eg, naloxone), were estimated to decrease fatal opioid overdoses by up to 37% (scenarios B and D). Interventions yielding reduced overdose rates specifically among persons with OUD not currently receiving treatment with MOUD demonstrated the largest reduction in both nonfatal and fatal opioid overdoses due to the high proportion of persons with OUD not receiving treatment with MOUD. While efforts to increase MOUD initiation and decrease OUD recurrence had limited association with fatal opioid overdoses over the short-term period simulated here (2021 and 2023), these interventions were associated with reducing OUD prevalence and increasing MOUD prevalence. Results of scenario A, which combined increasing MOUD initiation with decreasing OUD recurrence, estimated that OUD prevalence could be reduced by up to 23% and MOUD prevalence could be increased by up to 137%, although this required tripling the MOUD initiation rate over 3 years (from 9.3% to 27.9% among persons with OUD). Interventions with these goals may have a greater influence on fatal and nonfatal overdoses over a longer time horizon once a higher proportion of persons with OUD could be initiated into MOUD treatment. This highlights the need for interventions that increase awareness of OUD symptoms, clinician training for appropriate OUD screening and diagnosis, and efforts to reduce stigma so that persons with OUD can be appropriately linked to treatment.^6,7,30^

Decreasing OUD prevalence and increasing access to MOUD treatment are 2 important objectives of Healthy People 2030^31^ and are components of the US Department of Health and Human Services Overdose Prevention Strategy.^9^ Our findings suggest that, in the short-term, expansion of evidence-based interventions aiming to reduce risk of nonfatal and fatal overdose (eg, overdose education, naloxone distribution) are critical to achieve maximum reductions of fatal opioid overdoses. In the long-term, interventions aimed at increasing MOUD initiation, retention of persons in treatment, and recovery support are critical to reducing OUD prevalence and can potentially achieve further reductions in opioid overdoses. CDC’s strategic priorities in the Division of Overdose Prevention^32^ support public health interventions designed to achieve these objectives through programs such as Overdose Data to Action^33^ and the 2022 CDC Clinical Practice Guideline for Prescribing Opioids for Pain.^34^ Examples include supporting capacity building at state, local, and community levels, increased MOUD provision and access, and naloxone distribution. Increasing MOUD access may occur through a variety of linkage to care and retention mechanisms, such as decreasing the time to access and be linked to treatment, increased prescribing and availability of MOUD, and implementation of peer support programs and efforts to address stigma.^6,7,35^ Efforts to decrease both nonfatal and fatal opioid-involved overdoses can encompass overdose education and harm reduction efforts including increased access to and distribution of naloxone and drug-checking services.^14,36,37^

### Limitations

This study has several limitations. First, the model does not simulate the impacts of specific intervention programs among persons with OUD due to limited data. We also cannot comment on the investments or intensity of efforts necessary to achieve the change in the magnitude of hypothetical interventions simulated here. Second, this model does not distinguish between type of opioid involved in overdoses. IMF is increasingly involved in overdose deaths, and while the data used implicitly account for increases in overdose deaths due to IMF, our projections do not account for future changes in lethality through increases in fentanyl within the drug supply.^2,38,39,40^ Furthermore, we obtained fatal overdose rate estimates among persons receiving MOUD from a meta-analysis of studies^41^ published before IMF driving increasing mortality rates. As a result, it is possible that our analysis underestimates the rate of fatal overdoses among persons in MOUD treatment and overestimates fatal overdose rates among persons with OUD not receiving MOUD treatment. Third, MODIPHI is an aggregate model and cannot track individual histories. As a result, the model does not differentiate probability of treatment initiation and receipt based on history of MOUD. Furthermore, the model does not account for the increased risk of fatal overdose among persons with past nonfatal overdoses.^42,43^ The validation of nonfatal overdoses utilized 1 estimate among people who inject drugs, which may not be representative of people with OUD. Accurately tracking nonfatal overdoses is limited by their timely identification and lack of data sources linking nonfatal and fatal overdoses at the individual level.^44,45^ Fourth, due to limitations in data quality, comparability, and availability, we used 2019 to 2020 data to calibrate the model and could not project outcomes over a longer time horizon, which could reveal additional dynamics. Furthermore, the COVID-19 pandemic had impacts on data collection as well as health care access and utilization during 2020 and the projected 2021 to 2023 period, which may affect these results in unknown ways. However, we conducted sensitivity analyses on the 2020 calibration target of OUD prevalence reported in NSDUH and found low sensitivity, with identical results to those reported in our scenario analyses (eAppendix 14, eTable 12, and eFigure 9 in Supplement 1). Fifth, due to sample size constraints in the MOUD Study, the model cannot distinguish between specific MOUD treatments. Furthermore, responses in the MOUD Study were self-reported and subject to social desirability bias, excluded certain age groups in our study (ie, those aged 12-17 years), and excluded information about those who may have been lost to follow-up, dropped out of treatment, or died. Sixth, the study population represents persons with OUD, a patient population who are likely underdiagnosed or underestimated.^22,28^ Additionally, proxy variables were used in SUDORS to identify possible OUD among opioid-involved overdose decedents.^25^ Despite this limitation, sensitivity analysis of the proportion of decedents with prior OUD in the SUDORS data showed our results to be robust (eAppendix 15 and eTable 13 in Supplement 1). Additionally, MODIPHI only represents persons with OUD and the model does not account for any future interventions that might reduce the incidence of OUD.

## Conclusions

This decision analytical model study provides insight about the population dynamics of MOUD treatment and opioid overdose among persons with OUD and the association between hypothetical outcomes of combined public health interventions and opioid overdose-related outcomes nationally. Findings suggest that expansion of evidence-based interventions to reduce the risk of overdose fatality among persons with OUD, such as through harm reduction efforts (eg, overdose education or naloxone distribution) are critical to achieve maximal reductions in fatal opioid-involved overdoses in the short-term. These results also emphasize that efforts to increase MOUD initiation and retain persons in treatment (eg, linkage to care, low-barrier treatment, or behavioral interventions) engender marked improvement in MOUD and OUD prevalence but may have limited influence on fatal opioid-involved overdoses in the short-term. A multifaceted and multisector approach that includes collaborations across health systems, public health, public safety, and community-based organizations will be important to successfully implement and scale up this comprehensive suite of interventions required to both reduce opioid-involved overdose fatalities in the short-term and sustain improved outcomes over the long-term.

## Notes

Supplement 1. **eAppendix 1.** Model Structure **eAppendix 2. **Summary of Model Parameters **eTable 1.** Model Input Parameters, Static Unless Specified Otherwise **eAppendix 3. **MOUD Study **eAppendix 4. **IBM MarketScan Claims Database **eTable 2.** Diagnosis, Procedure, and National Drug Codes (NDCs) Used Within MarketScan Treatment Pathways to Identify Individuals With OUD, MOUD, and Opioid Involved Overdose **eTable 3.** Identifying Proportion of Individuals With OUD not Currently Receiving MOUD who Experienced an Overdose From the MarketScan Commercial and Medicaid Databases (2018-2021) **eAppendix 5. **Literature Data Sources **eTable 4.** Model Initialization Parameters **eAppendix 6. **Model Initialization **eTable 5.** Model Calibration Targets **eAppendix 7. **Calibrating to Fatal Overdoses **eTable 6.** Estimation of Proportion of Decedents who Died From an Opioid-Involved Overdose With Prior Opioid use Disorder Using Data From the State Unintentional Drug Overdose Reporting System (SUDORS) in 26 Jurisdictions, 2019 to 2020 **eTable 7.** Estimation of Number of National Overdoses Among Individuals With Opioid use Disorder (OUD) Using Data From the State Unintentional Drug Overdose Reporting System (SUDORS) and National Vital Statistics System (NVSS) Mortality Data, 2019 to 2020 **eAppendix 8. **Calibrating to Overall OUD Prevalence **eAppendix 9. **Calibrating to MOUD Prevalence **eAppendix 10. **Calibration Results **eTable 8.** Calibrated Parameter Ranges and Calibrated Values **eFigure 1. **Calibration Results Showing Pre- and Postcalibrated Model Outcomes in Relation to Targets **eAppendix 11. **Validating Nonfatal Overdoses and Reporting Overdoses **eAppendix 12. **Multiple Intervention Scenarios **eTable 9.** Summary of Annual Model Outcomes Under the Baseline Scenario **eTable 10.** Model Outcomes for Baseline and Intervention Scenarios When Interventions are set to the Maximum Effect Size Modeled. Outcomes Reported at end of Model Simulation (end of 2023) **eTable 11.** Summary of Relative Impact on Model Outcomes Across all Multiple Intervention Scenarios **eFigure 2.** Comparison of Model Outcomes From Scenarios A to D Over Time Relative to Baseline Scenario **eAppendix 13. **One-Way Parameter Sensitivity Analysis **eFigure 3. **One-Way Sensitivity Analysis Showing Association Between Parameter Perturbations Ranging From −100% to 200% and Fatal Overdoses Relative to Baseline Scenario **eFigure 4. **One-Way Sensitivity Analysis Showing Association Between Parameter Perturbations Ranging From −100% to 200% and Nonfatal Overdoses Relative to Baseline Scenario **eFigure 5.** One-Way Sensitivity Analysis Showing Association Between Parameter Perturbations Ranging From −100% to 200% and Number of Individuals With OUD not Receiving MOUD (OUD Prevalence) Relative to Baseline Scenario **eFigure 6.** One-Way Sensitivity Analysis Showing Association Between Parameter Perturbations Ranging From −100% to 200% and Number of Individuals Receiving MOUD (MOUD Prevalence) Relative to Baseline Scenario **eFigure 7.** One-way Sensitivity Analysis Showing Association Between Parameter Perturbations Ranging From −100% to 200% and Number of Individuals in Remission Relative to Baseline Scenario **eFigure 8.** One-way Sensitivity Analysis Showing Association Between Parameter Perturbations Ranging From −100% to 200% and Deaths From Other Causes Relative to Baseline Scenario **eAppendix 14. **Sensitivity Analysis of 2020 OUD Prevalence Data **eTable 12.** Summary of Annual Model Outcomes Under the Baseline Scenario Given Sensitivity Analysis of 2020 OUD Prevalence as a Calibration Target **eFigure 9.** Percentage Change in Projected Model Outcomes Relative to the Baseline Scenario for Fatal Overdoses, Nonfatal Overdoses, OUD Prevalence (Without MOUD) and MOUD Prevalence at the end of 2023 **eAppendix 15. **Sensitivity Analysis of SUDORS Data **eTable 13.** Summary of Annual Model Outcomes Under the Baseline Scenario Given Sensitivity Analysis of Increases in SUDORS Proportion of Decedents With Opioid-Involved Overdoses and Prior OUD
eReferences

Supplement 2.
Data Sharing Statement
