# Supplementary material for: Public Health Interventions and Overdose-Related Outcomes Among Persons With Opioid Use Disorder
Source: JAMA Netw Open. 2024 Apr 3;7(4):e244617. doi: 10.1001/jamanetworkopen.2024.4617 (PMC10993074; doi:10.1001/jamanetworkopen.2024.4617)
Supplement: Supplement 1. — eAppendix 1. Model Structure eAppendix 2. Summary of Model Parameters eTable 1. Model Input Parameters, Static Unless Specified Otherwise eAppendix 3. MOUD Study eAppendix 4. IBM MarketScan Claims Database eTable 2. Diagnosis, Procedure, and National Drug Codes (NDCs) Used Within MarketScan Treatment Pathways to Identify Individuals With OUD, MOUD, and Opioid Involved Overdose eTable 3. Identifying Proportion of Individuals With OUD not Currently Receiving MOUD who Experienced an Overdose From the MarketScan Commercial and Medicaid Databases (2018-2021) eAppendix 5. Literature Data Sources eTable 4. Model Initialization Parameters eAppendix 6. Model Initialization eTable 5. Model Calibration Targets eAppendix 7. Calibrating to Fatal Overdoses eTable 6. Estimation of Proportion of Decedents who Died From an Opioid-Involved Overdose With Prior Opioid use Disorder Using Data From the State Unintentional Drug Overdose Reporting System (SUDORS) in 26 Jurisdictions, 2019 to 2020 eTable 7. Estimation of Number of National Overdoses Among Individuals With Opioid use Disorder (OUD) Using Data From the State Unintentional Drug Overdose Reporting System (SUDORS) and National Vital Statistics System (NVSS) Mortality Data, 2019 to 2020 eAppendix 8. Calibrating to Overall OUD Prevalence eAppendix 9. Calibrating to MOUD Prevalence eAppendix 10. Calibration Results eTable 8. Calibrated Parameter Ranges and Calibrated Values eFigure 1. Calibration Results Showing Pre- and Postcalibrated Model Outcomes in Relation to Targets eAppendix 11. Validating Nonfatal Overdoses and Reporting Overdoses eAppendix 12. Multiple Intervention Scenarios eTable 9. Summary of Annual Model Outcomes Under the Baseline Scenario eTable 10. Model Outcomes for Baseline and Intervention Scenarios When Interventions are set to the Maximum Effect Size Modeled. Outcomes Reported at end of Model Simulation (end of 2023) eTable 11. Summary of Relative Impact on Model Outcomes Across all Multiple Intervention Scena [file jamanetwopen-e244617-s001.pdf]

Supplemental Online Content

Nataraj N, Rikard SM, Zhang K, et al. Public health interventions and overdose-related outcomes among persons with opioid use disorder. *JAMA Netw Open*. 2024;7(4):e244617. doi:10.1001/jamanetworkopen.2024.4617

eAppendix 1. Model Structure
eAppendix 2. Summary of Model Parameters
eTable 1. Model Input Parameters, Static Unless Specified Otherwise
eAppendix 3. MOUD Study
eAppendix 4. IBM MarketScan Claims Database
eTable 2. Diagnosis, Procedure, and National Drug Codes (NDCs) Used Within MarketScan Treatment Pathways to Identify Individuals With OUD, MOUD, and Opioid Involved Overdose
eTable 3. Identifying Proportion of Individuals With OUD not Currently Receiving MOUD who Experienced an Overdose From the MarketScan Commercial and Medicaid Databases (2018-2021)
eAppendix 5. Literature Data Sources
eTable 4. Model Initialization Parameters
eAppendix 6. Model Initialization
eTable 5. Model Calibration Targets
eAppendix 7. Calibrating to Fatal Overdoses
eTable 6. Estimation of Proportion of Decedents who Died From an Opioid-Involved Overdose With Prior Opioid use Disorder Using Data From the State Unintentional Drug Overdose Reporting System (SUDORS) in 26 Jurisdictions, 2019 to 2020
eTable 7. Estimation of Number of National Overdoses Among Individuals With Opioid use Disorder (OUD) Using Data From the State Unintentional Drug Overdose Reporting System (SUDORS) and National Vital Statistics System (NVSS) Mortality Data, 2019 to 2020
eAppendix 8. Calibrating to Overall OUD Prevalence
eAppendix 9. Calibrating to MOUD Prevalence
eAppendix 10. Calibration Results
eTable 8. Calibrated Parameter Ranges and Calibrated Values
eFigure 1. Calibration Results Showing Pre- and Postcalibrated Model Outcomes in Relation to Targets
eAppendix 11. Validating Nonfatal Overdoses and Reporting Overdoses
eAppendix 12. Multiple Intervention Scenarios
eTable 9. Summary of Annual Model Outcomes Under the Baseline Scenario
eTable 10. Model Outcomes for Baseline and Intervention Scenarios When Interventions are set to the Maximum Effect Size Modeled. Outcomes Reported at end of Model Simulation (end of 2023)
eTable 11. Summary of Relative Impact on Model Outcomes Across all Multiple Intervention Scenarios
eFigure 2. Comparison of Model Outcomes From Scenarios A to D Over Time Relative to Baseline Scenario
eAppendix 13. One-Way Parameter Sensitivity Analysis
eFigure 3. One-Way Sensitivity Analysis Showing Association Between Parameter Perturbations

© 2024 Nataraj N et al. *JAMA Network Open.*

Ranging From −100% to 200% and Fatal Overdoses Relative to Baseline Scenario
eFigure 4. One-Way Sensitivity Analysis Showing Association Between Parameter Perturbations Ranging From −100% to 200% and Nonfatal Overdoses Relative to Baseline Scenario
eFigure 5. One-Way Sensitivity Analysis Showing Association Between Parameter Perturbations Ranging From −100% to 200% and Number of Individuals With OUD not Receiving MOUD (OUD Prevalence) Relative to Baseline Scenario
eFigure 6. One-Way Sensitivity Analysis Showing Association Between Parameter Perturbations Ranging From −100% to 200% and Number of Individuals Receiving MOUD (MOUD Prevalence) Relative to Baseline Scenario
eFigure 7. One-way Sensitivity Analysis Showing Association Between Parameter Perturbations Ranging From −100% to 200% and Number of Individuals in Remission Relative to Baseline Scenario
eFigure 8. One-way Sensitivity Analysis Showing Association Between Parameter Perturbations Ranging From −100% to 200% and Deaths From Other Causes Relative to Baseline Scenario
eAppendix 14. Sensitivity Analysis of 2020 OUD Prevalence Data
eTable 12. Summary of Annual Model Outcomes Under the Baseline Scenario Given Sensitivity Analysis of 2020 OUD Prevalence as a Calibration Target
eFigure 9. Percentage Change in Projected Model Outcomes Relative to the Baseline Scenario for Fatal Overdoses, Nonfatal Overdoses, OUD Prevalence (Without MOUD) and MOUD Prevalence at the end of 2023
eAppendix 15. Sensitivity Analysis of SUDORS Data
eTable 13. Summary of Annual Model Outcomes Under the Baseline Scenario Given Sensitivity Analysis of Increases in SUDORS Proportion of Decedents With Opioid-Involved Overdoses and Prior OUD
eReferences

This supplemental material has been provided by the authors to give readers additional information about their work.

eAppendix 1. Model structure

Model states

The OUD natural history model was developed in consultation with all coauthors and additional subject matter experts in substance use disorder. The model consists of the following states, defined below:

- Opioid use disorder (OUD): Individuals who have a past year OUD without current receipt of medications for opioid use disorder (MOUD) as identified by DSM-IV or DSM-5 criteria used in the 2018-2020 National Survey on Drug Use and Health (NSDUH) annual reports[1-3].
- Receipt of MOUD for ≤1 month, >1-6 months, >6-12 months, and >12 months: Individuals who are receiving MOUD (buprenorphine, methadone, or naltrexone, with or without counseling), stratified by duration of time in treatment.
- Remission: Remission from OUD is often defined as the absence of DSM-5 OUD criteria symptoms[4-6], however, we are unable to identify all criteria symptoms in our data sources. Additionally, remission may be described as early or sustained based on duration of abstinence from opioids, however, our focus in this paper is on sustained remission (≥ 12 months) achieved through either MOUD or non-MOUD treatment[4]. Therefore, we define remission based on duration of time in treatment *and* abstinence from opioid use as: no reported opioid use for the past 90 days and receipt of treatment with either MOUD or non-MOUD counseling-only services for at least 360 days. We note that, in our model, individuals who have achieved remission after 360 days with MOUD treatment may or may not continue to receive MOUD thereafter.
- Fatal opioid-involved overdose: Individuals with OUD (with or without receipt of MOUD) who died of an opioid-involved overdose.
- Nonfatal opioid-involved overdose: Individuals with OUD receiving MOUD who self-reported recent nonfatal overdose in the MOUD Study[7] or those without MOUD with administrative claims indicating an opioid-involved overdose, identified via a combination of diagnoses, procedural, and national drug codes available in Xu et al (2022), eTable.1[8].
- Death from other causes: Individuals with OUD (with or without current receipt of MOUD) who died from causes other than an opioid overdose.

Study population

The population of interest in this study is individuals aged ≥12 years with OUD. Prevalence estimates of OUD for 2018 (used to initialize the model on 1/1/2019) and 2019 (used as a calibration target) were obtained from the 2018 and 2019 NSDUH Annual Reports[1,2], adjusted for national prevalence via the benchmark multiplier method based on data from the Transformed Medicaid Statistical Information System (T-MSIS) as available in eTable 2, Mojtabai (2022)[9]. We note that these estimates include individuals receiving MOUD treatment.

© 2024 Nataraj N et al. *JAMA Network Open.*

eAppendix 2. Summary of Model Parameters

Model parameters were identified using a combination of literature sources and data from NSDUH, the MOUD Study, and MarketScan® Commercial and Multi-State Medicaid Databases. Parameter values used in the calibrated model are specified in eTable 1.

eTable 1. Model Input Parameters, Static Unless Specified Otherwise.

Parameter	Value	Units	Source
**Annual number of individuals newly diagnosed with OUD (2019, 2020)**	Y2019 = 2,914,637<br>Y2020 = 2,893,069	Individuals/year	*Calibrated (See eTable 5)*
**Annual proportion of individuals who initiate MOUD each year (2019, 2020)**	Y2019 = 0.225<br>Y2020 = 0.102	Annual probability	*Calibrated (See eTable 5)*
**Proportion of individuals with OUD receiving counseling only treatment**	0.099	Annual probability	*Calibrated (See eTable 5)*
**Proportion of individuals receiving counseling only treatment who achieve remission**	0.0491	Annual probability	MOUD Study
Transitions to OUD (i.e., recurrence) from each of the following states:			
**MOUD ≤1 month**	0.3873	Annual probability	MOUD Study
**MOUD >1-6 months**	0.2278	Annual probability	MOUD Study
**MOUD >6-12 months**	0.1864	Annual probability	MOUD Study
**MOUD >12 months**	0.1605	Annual probability	MOUD Study
**Remission**	0.0088	Annual probability	MOUD Study
Transitions to nonfatal overdose from each of the following states:			
**OUD**	0.1136	Annual probability	MarketScan Medicaid and Commercial Claims Databases
**MOUD ≤1 month**	0.0110	Annual probability	Literature[10]
**MOUD >1-6 months**	0.0559	Annual probability	MOUD Study
**MOUD >6-12 months**	0.0410	Annual probability	MOUD Study
**MOUD >12 months**	0.0701	Annual probability	MOUD Study
**Remission**	0.0123	Annual probability	MOUD Study
Transitions to fatal overdose from each of the following states:			
**OUD, annually (2019, 2020)**	Y2019 = 0.00271<br>Y2020 = 0.00291	Annual probability	*Calibrated (See eTable 5)*
**MOUD ≤1 month**	0.00220	Annual probability	Literature[11]
**MOUD >1-6 months**	0.00175	Annual probability	Literature[11]
**MOUD >6-12 months**	0.00175	Annual probability	Literature[11]
**MOUD >12 months**	0.00175	Annual probability	Literature[11]
**Remission**	0.00175	Annual probability	Literature[11]
Transitions to death from other causes from each of the following states:			
**OUD**	0.01415	Annual probability	Literature[11]
**MOUD ≤1 month**	0.00575	Annual probability	Literature[11]
**MOUD >1-6 months**	0.00330	Annual probability	Literature[11]
**MOUD >6-12 months**	0.00330	Annual probability	Literature[11]
**MOUD >12 months**	0.00330	Annual probability	Literature[11]
**Remission**	0.00330	Annual probability	Literature[11]

© 2024 Nataraj N et al. *JAMA Network Open.*

eAppendix 3. MOUD Study

We parameterized several transitions related to MOUD outcomes including recurrence, nonfatal overdose, and remission based on reported duration of treatment using the MOUD Study[7]. We also used the study to parameterize the proportion of individuals reporting counseling only treatment (i.e., non-MOUD treatment) who achieved remission. We used responses from baseline (i.e., study enrollment), 3-,6-, 12-, and 18-month questionnaires provided to patients receiving OUD treatment from 62 participating OUD outpatient treatment facilities between March 2018-May 2021. Types of treatment facilities included facilities focusing primarily on substance use services, both mental health and substance use, or general health care.

The specific OUD treatment a patient was receiving when screened for study eligibility is referred to as the index treatment. For individuals receiving MOUD, we restricted the analysis to individuals reporting index buprenorphine, methadone, or naltrexone treatment and excluded those who reported index treatment of counseling alone. At baseline assessment, individuals may have been receiving index treatment for a specified duration already, identified via the length of treatment variable. We stratified individuals based on time in treatment for each questionnaire and then used a weighted average of responses across questionnaires when parameterizing the model. This analysis used individual-level characteristics at each assessment including length of index treatment, status of index treatment indicating completion or cessation for a variety of reasons, past 30- and 90-day opioid use, and past 90-day opioid overdose, to identify the following outcomes, stratified by duration of treatment, subsequently used to parameterize model transitions:

1. <u>Recurrence of OUD from MOUD:</u> Defined among individuals in MOUD who indicated no current receipt of MOUD treatment *and* past 30-day opioid use (for duration of treatment <30 days) or past 90-day opioid use (for all other durations of treatment). We assume that individuals who are still receiving MOUD but report recent opioid use do not experience recurrence. Previous studies have identified recurrence of use as 10 or more days of non-prescription opioid use in the past 4 weeks[12,13], however we could not determine number of days of opioid use in this study.
2. <u>Nonfatal overdose from MOUD:</u> Defined among individuals in MOUD who reported experiencing an opioid overdose in the past 90 days. We are limited by the availability of data only on past 90-day overdose – therefore, we use overdose-related data only from individuals who have been in treatment for at least 90 days to parameterize rates of nonfatal overdose among all individuals in the >1-6 months MOUD state.
3. <u>Remission through MOUD:</u> Defined among individuals receiving MOUD who indicated >360 days of MOUD index treatment and no past 90-day opioid use or overdose.
4. <u>Returning to >12 Month MOUD after remission:</u> Defined among individuals in remission who reported past 90-day opioid use while continuing to receive index

© 2024 Nataraj N et al. *JAMA Network Open.*

MOUD treatment. Since our population-level model does not account for history of MOUD among individuals in remission and our model allows for the possibility of individuals to directly enter remission in the absence of MOUD[14], we assume a single rate of return to MOUD for all individuals in remission, regardless of whether they previously received MOUD or not This assumption might result in slightly overestimating the number of individuals who receive MOUD after remission.

5. Recurrence of OUD from remission: Defined among individuals in remission who reported both past 90-day opioid use and that they are no longer receiving index MOUD treatment. As in the previous case, we assume a single rate of recurrence to OUD after remission among individuals with and without a history of MOUD. This could potentially result in an underestimate of OUD recurrence among individuals in remission, since this rate is informed entirely by individuals who have a history of MOUD.
6. Nonfatal overdose from remission: Defined among individuals in remission who reported experiencing an opioid overdose in the past 90 days.

We additionally used responses from individuals who reported receiving non-MOUD treatment, i.e., counseling-only treatment to help parameterize the following:

1. Proportion of individuals who receive counseling only treatment who achieve remission from OUD: Defined among individuals who indicated >360 days of counseling-only index treatment and no past 90-day opioid use or overdose.

eAppendix 4. IBM MarketScan Claims Database

To determine the rate of nonfatal overdose among individuals with OUD not currently receiving MOUD we used the MarketScan Treatment Pathways® platform. The MarketScan® Research Database contain individual level, deidentified healthcare claims information from employers, health plans, hospitals, and Medicare and Medicaid programs. These databases reflect real-world treatment patterns and costs by tracking millions of patients as they travel through the healthcare system, offering detailed information about all aspects of care. Data about individual patients are integrated from all providers of care, maintaining healthcare utilization and cost record connections at the patient level.

Among the 16,241,976 individuals within the multistate Medicaid database and the 58,791,825 individuals within the Commercial database aged ≥12 years on 1/1/2018, we identified, respectively, 449,427 and 204,358 individuals with an ICD-10-CM diagnosis of OUD (eTable 2) between 2018 and 2021. Individuals with at least 90 days of continuous enrollment following their OUD diagnosis were retained for further analysis. Among this continuously enrolled population, we identified individuals who experienced an overdose between the date of OUD diagnosis and 12/31/2021 (eTable 2) and no MOUD treatment prior to overdose (eTables 2 and 3). We then used the weighted average of the OUD population with Commercial and Medicaid

© 2024 Nataraj N et al. *JAMA Network Open.*

insurance who experienced opioid overdoses and average times to overdose to determine the rate of nonfatal overdose among persons with OUD.

eTable 2. Diagnosis, Procedure, and National Drug Codes (NDCs) Used Within MarketScan Treatment Pathways® to Identify Individuals With OUD, MOUD, and Opioid Involved Overdose

Category	Codes
**OUD: ICD-10-CM Diagnosis**	F1110;F1111;F11120; F11121; F11122; F11129; F1112; F1113; F1114; F11150; F11151; F11159; F1115; F11181; F11182; F11188; F1118; F1119; F111; F1120; F1121; F11220; F11221; F11222; F11229; F1122; F1123; F1124; F11250; F11251; F11259; F1125; F11281; F11282; F11288; F1128; F1129; F112
**Opioid Overdose: ICD-10-CM Diagnosis**	T400; T400X1; T400X1A; T400X1D; T400X1S; T400X2; T400X2A; T400X2D; T400X2S; T400X3; T400X3A; T400X3D; T400X3S; T400X4; T400X4A; T400X4D; T400X4S; T400X5; T400X5A; T400X5D; T400X5S; T400X; T401; T401X1; T401X1A; T401X1D; T401X1S; T401X2; T401X2A; T401X2D; T401X2S; T401X3; T401X3A; T401X3D; T401X3S; T401X4; T401X4A; T401X4D; T401X4S; T401X; T402; T402X1; T402X1A; T402X1D; T402X1S; T402X2; T402X2A; T402X2D; T402X2S; T402X3; T402X3A; T402X3D; T402X3S; T402X; T402X4A; T402X4D; T402X4S; T402X5; T402X5A; T402X5D; T402X5S; T402X; T403; T403X1; T403XA; T403X1D; T403X1S; T403X2; T403X2A; T403X2D; T403X2S; T403X3; T403X3A; T403X3D; T403X3S; T43X4; T403X4A; T403X4D; T403X4S; T403X5; T403X5A; T403X5D; T403X5S; T403X; T40411; T40411A; T40411D; T40411S; T40412; T40412A; T40412D; T40412S; T40413; T40413A; T40413D; T40413S; T40414; T40414A; T40414D; T40414S; T40415; T40415A; T40415D; T40415S; T4041; T40421; T40421A; T40421D; T40421S; T40422; T40422A; T40422D; T40422S; T40423; T40423A; T40423D; T40423S; T40424; T40424A; T40424D; T40424S; T40425; T40425A; T40425D; T40425S; T4042; T40491; T40491A; T40491D; T40491S; T40492; T40492A; T40492D; T40492S; T40493; T40493A; T40493D; T40493S; T40494; T40494A; T40494D; T40494S; T40495; T40495A; T40495D; T40495S; T4049; T404; T404X1; T404X1A; T404X1D; T404X1S; T404X; T404X2A; T404X2D; T404X2S; T404X3; T404X3A; T404X3D; T404X3S; T404X4; T404X4A; T404X4D; T404X4S; T404X5; T404X5A; T404X5D; T404X5S; T404X; T40601A; T40601; T40601D; T40601S; T40602; T40602A; T40602D; T40602S; T40603; T40603A; T40603D; T40603S; T40604; T40604A; T40604D; T40604S; T40605; T40605A; T40605D; T40605S; T4060; T40691; T40691A; T40691D; T40691S; T40692; T40692A; T40692D; T40692S; T40693; T40693A; T40693D; T40693S; T40694; T40694A; T40694D; T40694S; T40695; T40695A; T40695D; T40695S; T4069; T406
**MOUD: Procedure codes**	J2315; H2020
**MOUD: Buprenorphine NDCs**	54017613; 54017713; 54018813; 54018913; 93537856; 93537956; 93572056; 93572156; 228315303; 228315403; 228315473; 228315503; 228315573; 228315603; 378092393; 378092493; 406192303; 406192403; 490005130; 12496120201; 12496120203; 12496120401; 12496120403; 12496120801; 12496120803; 12496121201; 12496121203; 12496127802; 12496128302; 12496130602; 12496131002; 16590066630; 16590066730; 23490927003; 35356000430; 38779088800; 38779088801; 38779088803; 38779088809; 42291017430; 42291017530; 43063018430; 49452129203; 49452825301; 49452825302; 49452825303; 49452825304; 49999039515; 49999063830; 49999063930; 50383028793; 50383029493; 50383092493; 50383093093; 51927101200; 52959030430; 52959074930; 54123011430; 54123091430; 54123092930; 54123095730; 54123098630; 54569573900; 54569573901; 54569573902; 54569639900; 54868570700; 54868570701; 54868575000; 55700014730; 59385001201; 59385001230; 59385001401; 59385001430; 59385001601; 59385001630; 60429058630; 60429058730; 62991158301; 62991158302; 62991158303; 62991158304; 63275992202; 63370090506; 63370090509; 63370090510; 63370090515; 63874108503; 63874117303; 65162041503; 65162041603; 68071151003; 68308020830
**MOUD: Naltrexone NDCs**	56001130; 56001170; 185003901; 185003930; 406117001; 406117003; 555090201; 555090202; 16729008101; 16729008110; 38779088703; 38779088704; 38779088705; 38779088706; 42291063230; 47335032683; 47335032688; 49452483501; 49452483502; 51224020630; 51224020650; 51285027501; 51285027502; 51552073701; 51552073702; 51927354800; 51927360200; 51927437700; 52152010502; 52152010530; 52372075102; 62991124301; 62991124302; 62991124303; 62991124304; 63370015810; 63370015815; 63370015825; 63370015835; 68084029121; 68094085362; 63459030042; 65757030001

eTable 3. Identifying Proportion of Individuals With OUD not Currently Receiving MOUD who Experienced an Overdose From the MarketScan Commercial and Medicaid Databases (2018-2021)

MarketScan Database (2018-2021)	Population with OUD and no current MOUD (N)	Subset of patients with opioid overdoses (N)	Proportion experiencing opioid overdoses (%)	Average time between OUD Dx and first overdose, given no MOUD (days)
**Commercial**	126,253	4,706	3.73	209.76
**Multi-state Medicaid**	238,097	24,807	10.42	274.62
**Weighted average**			**8.10**	**252.14**

eAppendix 5. Literature Data Sources

Literature sources were used to identify rates of overdose mortality and other-cause mortality among individuals with OUD with and without MOUD treatment, as well as rates of nonfatal overdose among individuals receiving MOUD treatment for ≤30 days. To the best of our knowledge, no previous research distinguishes between overdose-related and other-cause mortality based on more granular categories of duration in MOUD treatment, thus, limiting our ability to incorporate declining mortality rates after the first month in treatment.

- Overdose mortality: A systematic review and meta-analysis by *Sordo et. al* included 19 cohort studies in a comparison of the risk for all-cause mortality and overdose mortality in people with opioid dependence during and after periods of treatment with methadone or buprenorphine.[11] They report the pooled overdose mortality rate for methadone and buprenorphine separately during the first four weeks and after four weeks in treatment and out of treatment. We averaged the pooled overdose mortality rates for the first four weeks in treatment for methadone and buprenorphine to define the rate of fatal overdose from the MOUD ≤1 month model state. We averaged the pooled overdose mortality rates after four weeks in treatment to define the rate of fatal overdose from the MOUD >1-6 months, MOUD >6-12 months, MOUD >12 months, and Remission model states. It is important to note that in these cohort studies, overdose mortality is not necessarily limited to opioid-involved overdoses, potentially overestimating overdose mortality rate among individuals receiving MOUD in our model of opioid-involved overdoses.
- Other-cause mortality: Similarly, *Sordo et. al* report pooled all-cause mortality rates separately for methadone and buprenorphine during the first four weeks and after four weeks in and out of treatment. To determine mortality rates due to all causes other than overdose mortality, we subtracted the overdose mortality rates from all-cause mortality rates. We averaged the pooled other-cause mortality rates for periods out of treatment to define the rate of other-cause deaths for the OUD model state. We averaged the pooled other-cause mortality rates during the first four weeks in treatment to define the rate of other-cause deaths from the MOUD ≤1 month model state and averaged the pooled other-cause mortality rates after four weeks in treatment to define the rate of other-cause deaths from the MOUD >1-6 months, MOUD >6-12 months, MOUD >12 months, and Remission model states.

- Nonfatal overdose receiving MOUD treatment for ≤1 month: Since the MOUD Study does not capture history of overdose under 90 days, we could not accurately identify rates of nonfatal overdose for individuals receiving MOUD ≤1 month. Instead, we used a study by Wakeman et. al (2020)[10] that uses claims data (OptumLabs Data Warehouse) to identify opioid-involved overdose among individuals with OUD in six unique treatment pathways (including buprenorphine or methadone). We use the probability of opioid overdose at 30 days among individuals receiving buprenorphine or methadone to define the rate of nonfatal overdose from the MOUD ≤1 Month model state.

eTable 4. Model Initialization Parameters

Parameter	Description	Value	Source
**$OUD_{initial}$**	Initial population of individuals with OUD, including those receiving MOUD	8,640,000 individuals	NSDUH 2018 Report, adjusted[15]
**$p\_MOUD_{initial}$**	Initial proportion of individuals receiving treatment for OUD	0.197	NSDUH 2018 Report
Among individuals receiving MOUD, initial proportion in each of the following model states			
**p_MOUD_{1month}**	MOUD ≤1 month	0.2719	MOUD Study
**$p\_MOUD_{>1\text{-}6month}$**	MOUD >1-6 months	0.3026	MOUD Study
**$p\_MOUD_{>6\text{-}12month}$**	MOUD >6-12 months	0.0996	MOUD Study
**$p\_MOUD_{12+month}$**	MOUD >12 months	0.3259	MOUD Study

eAppendix 6. Model Initialization

Parameters used to specify initial model states are described in eTable 4. Equations 1-5 were used to determine the initial populations of the model states for OUD, MOUD ≤1 month, MOUD >1-6 months, MOUD >6-12 months, and MOUD >12 months, where the prefix p_ indicates the proportion of individuals in each category. As shown in Equation 1, the OUD state excludes individuals with MOUD (i.e., $p\_MOUD_{initial}$) – these individuals are distributed across the four MOUD states at model initialization informed by the MOUD Study.

$$n\_OUD = OUD_{initial} * (1 - p\_MOUD_{initial}) \quad [1]$$

$$n\_MOUD \leq 1\text{ month} = OUD_{initial} * p\_MOUD_{initial} * p\_MOUD_{1month} \quad [2]$$

$$n\_MOUD > 1\text{-}6\text{ months} = OUD_{initial} * p\_MOUD_{initial} * p\_MOUD_{>1\text{-}6month} \quad [3]$$

$$n\_MOUD > 6\text{-}12\text{ months} = OUD_{initial} * p\_MOUD_{initial} * p\_MOUD_{>6\text{-}12month} \quad [4]$$

$$n\_MOUD > 12\text{ months} = OUD_{initial} * p\_MOUD_{initial} * p\_MOUD_{12+month} \quad [5]$$

The model states for remission, fatal overdose, nonfatal overdose, and death from other causes were initialized with a population of zero.

© 2024 Nataraj N et al. *JAMA Network Open.*

The model was calibrated to the following targets: Fatal overdoses, OUD (including MOUD) prevalence, and MOUD prevalence, described in more detail below.

eTable 5. Model Calibration Targets

Model outcome	2019 calibration target	2020 calibration target	Source
**Fatal overdoses**	24,796	58,927	SUDORS and NVSS
**OUD including MOUD Prevalence***	10,391,000	12,076,000	[2,3,9,16]
**MOUD Prevalence**†	1,880,771	1,352,512	[2,3,9,16]

*OUD Prevalence includes the following model states: OUD, MOUD ≤1 month, MOUD 1-6 months, MOUD >6-12 months, MOUD >12 months

†MOUD Prevalence includes the following model states: MOUD ≤1 month, MOUD >1-6 months, MOUD >6-12 months, MOUD >12 months

eAppendix 7. Calibrating to Fatal Overdoses

We used data from CDC's State Unintentional Drug Overdose Reporting System (SUDORS) to identify the likelihood of prior OUD among all individuals who experienced a fatal opioid-involved overdose (based on information from the death certificate or medical examiner or coroner reports indicating an opioid caused death), i.e., those who had evidence of current or past prescription opioid or heroin use, or treatment for substance use disorder (SUD) (eTable 6). This analysis included 26 jurisdictions that collected medical examiner and coroner reports from at least 75% of decedents during 2019 to 2020: Alaska, Connecticut, Delaware, Georgia, Illinois, Kentucky, Maine, Massachusetts, Minnesota, Missouri, Nevada, New Hampshire, New Jersey, New Mexico, North Carolina, Ohio, Oklahoma, Pennsylvania, Rhode Island, Tennessee, Utah, Vermont, Virginia, Washington, West Virginia, and the District of Columbia.

Since data from SUDORS is not nationally representative, we used the likelihood of opioid use disorder among individuals with fatal opioid overdoses applied to national estimates of fatal overdoses involving opioids obtained from the National Vital Statistics System (NVSS)[17] to obtain the calibration target of fatal opioid overdoses among individuals with prior OUD (eTable 7). This approach allowed us to leverage strengths of both surveillance systems. SUDORS captures detailed information from coroners and medical examiner (CME) reports around the circumstances surrounding the fatal drug overdose (including medical history, substance use disorder treatment history, postmortem toxicology results of all drugs detected and those determined to cause death) that is not available in the NVSS data, while the NVSS data serves to provide a national estimate of the number of fatal overdoses.

We note that some additional limitations of the SUDORS data include that our analysis was limited to jurisdictions that had CME reports available for at least 75% of deaths and to decedents with an available CME report. Further, circumstances represent evidence available in source documents and information provided in death investigations may vary; these are likely underestimated as death investigators might have limited information and negative responses may be indicative of no use disorder or that there was use disorder but not enough information to

justify coding that way. Despite this, sensitivity analysis presented in eAppendix 15 shows low sensitivity of scenario results to an assumed 25% increase in proportion of decedents with prior OUD. Additionally, we did not explicitly limit our analysis of SUDORS data to persons aged ≥12 years since there were few opioid overdose deaths reported for those <12 years old to influence the proportion of decedents with prior OUD[18]. Finally, to account for changes in variables collected in the SUDORS system and known data quality issues during the period of analysis, we combined data from 2019 and 2020 to determine the average proportion of decedents with prior OUD.

eTable 6. Estimation of Proportion of Decedents who Died From an Opioid-Involved Overdose With Prior Opioid use Disorder Using Data From the State Unintentional Drug Overdose Reporting System (SUDORS)in 26 Jurisdictions, 2019 to 2020.

SUDORS Variable	Data period				Aggregated	
	1/1/2019 – 6/30/2019	7/1/2019 – 12/31/2019	1/1/2020 – 6/30/2020	7/1/2020 – 12/31/2020	2019	2020
**Decedents with an opioid overdose**	12,497	13,869	17,100	16,942	26,366	34,042
Current or past prescription opioid misuse, heroin use, or treatment for a substance use disorder **[N]**	7,941	7,253	7,429	6,813	15,194	14,242
Current or past prescription opioid misuse, heroin use, or treatment for a substance use disorder **[%]**	63.54%	52.30%	43.44%	40.21%	57.63%	41.84%

eTable 7. Estimation of Number of National Overdoses Among Individuals With Opioid use Disorder (OUD) Using Data From the State Unintentional Drug Overdose Reporting System (SUDORS) and National Vital Statistics System (NVSS) Mortality Data, 2019 to 2020.

Year	SUDORS estimated proportion of decedents with prior OUD (2019-2020 average proportion)	NVSS fatal opioid overdoses (N)	Estimated opioid overdoses among individuals with OUD (N)
**2019**	0.49732	49,860	24,796
**2020**	0.49732	68,630	34,131
**Total**		118,490	58,927

eAppendix 8. Calibrating to Overall OUD Prevalence

Overall prevalence of OUD in 2019 and 2020, including individuals receiving MOUD, were used as calibration targets, using adjusted NSDUH prevalence estimates[2,3,9]. Given NSDUH updated the 2020 questionnaire to reflect Diagnostic and Statistical Manual of Mental Disorders-5 (DSM-5) categorization of opioid use disorder, it was not possible to directly compare prevalence estimates of OUD in 2020 directly with 2018-2019 data. To address this, we further adjusted 2020 DSM-5 prevalence estimates to reflect those corresponding to DSM-IV per published estimates[19]. We first determined the ratio of DSM-IV to DSM-5 population estimates of OUD based on published literature[19] that used data from the National Epidemiologic Survey on Alcohol and Related Conditions–III (NESARC-III) to be 0.88 for opioid and heroin use disorder combined. We then used this ratio to approximate DSM-IV estimates of overall OUD prevalence based on the prevalence reported in the 2020 NSDUH annual report[3] (2,700,000*0.88 = 2,378,772). Finally, we calculated the ratio of the OUD prevalence estimates as determined through the benchmark multiplier method[9] to those reported in NSDUH[1,2], averaged across 2018 and 2019 (determined to be 5.08), as a multiplier for the 2020 DSM-IV estimate of OUD prevalence we obtained in the previous step (2,378,772*5.08 = 12,076,158). It is worth noting that recent comparisons found high agreement between DSM-5 and DSM-IV based prevalence of opioid and heroin use disorder (Cohen's Kappa scores of 0.96 and 0.99 respectively)[20].

eAppendix 9. Calibrating to MOUD Prevalence

Estimates of the proportion of the population with OUD aged ≥12 years receiving MOUD from the 2019-2020 NSDUH annual reports[2,3] were multiplied by the adjusted overall OUD prevalence (including MOUD)[9] to obtain the estimated prevalence of individuals receiving MOUD in 2019 and 2020.

eAppendix 10. Calibration Results

Model calibration was performed by minimizing the sum of squared error (SSE) between model estimates and calibration targets described in eTable 5 at the time points t_1 = 12/31/2019, and t_2 = 12/31/2020. The objective function for calibration is described as follows:

$$\begin{aligned} SSE = \; & w_1 * ((FatalOD_{model,t_1} - FatalOD_{data,t_1})^2 + (FatalOD_{model,t_2} - FatalOD_{data,t_2})^2) + \\ & w_2 * ((OUD_{model,t_1} - OUD_{data,t_1})^2 + (OUD_{model,t_2} - OUD_{data,t_2})^2) + \\ & w_3 * ((MOUD_{model,t_1} - MOUD_{data,t_1})^2 + (MOUD_{model,t_2} - MOUD_{data,t_2})^2) \end{aligned} \quad [6]$$

where w_1 = 312, w_2 = 1, and w_3 = 7.2. These weights were used to scale error terms for each of the calibration targets to be on a similar scale of magnitude and were determined by dividing overall OUD prevalence (the highest value) by each of the calibration target values and averaging those respective results across 2019 and 2020. Outcomes with a "*model*" subscript denote model estimates at the specified time point, and outcomes with a *"data"* subscript denote calibration target data for the corresponding time point. Calibrated parameters were allowed to vary in the ranges provided in eTable 8. Optimization was performed using the OptQuest

© 2024 Nataraj N et al. *JAMA Network Open.*

optimizer engine with 50,000 model simulations. Model outcome estimates prior to calibration and post-calibration are provided in eFigure 1.

eTable 8. Calibrated Parameter Ranges and Calibrated Values

Parameter	Calibration Range Min	Calibration Range Max	Units	Calibrated Value
**Individuals newly diagnosed with OUD**	500,000	3,000,000	Individuals/year	Y2019 = 2,914,637<br>Y2020 = 2,893,069
**Proportion of individuals who initiate MOUD each year**	0.05	0.4	Annual probability	Y2019 = 0.225<br>Y2020 = 0.102
**Proportion of individuals with OUD with counseling only treatment**	0.08	0.22	Annual probability	0.099
**Transition from OUD to fatal overdose**	0.001	0.02	Annual probability	Y2019 = 0.00271<br>Y2020 = 0.00291

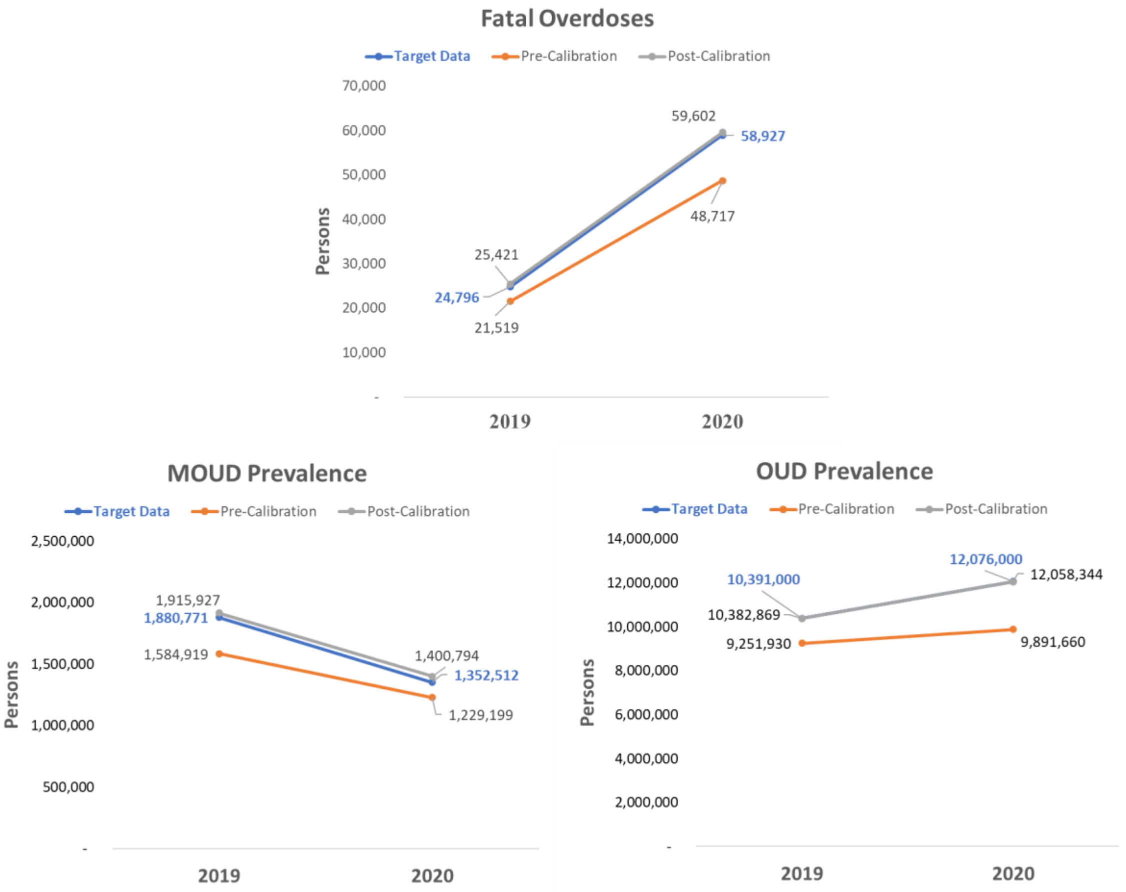

eFigure 1. Calibration Results Showing Pre- and Postcalibrated Model Outcomes in Relation to Targets.
*Overall OUD Prevalence includes the following model states: OUD, MOUD ≤1 month, MOUD >1-6 months, MOUD >6-12 months, MOUD >12 months
†MOUD Prevalence includes the following model states: MOUD ≤1 month, MOUD >1-6 months, MOUD >6-12 months, MOUD >12 months

© 2024 Nataraj N et al. *JAMA Network Open.*

eAppendix 11. Validating Nonfatal Overdoses and Reporting Overdoses

While there are several potential sources of data that capture nonfatal overdoses including emergency departments (ED) (administrative data and syndromic surveillance data), emergency medical services (EMS), poison control centers, law enforcement, and administrative claims, each of these has limitations, and we are limited in our ability to appropriately calibrate the model to the total number of national nonfatal overdoses[21,22]. Further, certain nonfatal overdoses may never be captured in traditional surveillance data since they may not present to a health care provider or even be witnessed. Therefore, most data sources likely underestimate nonfatal opioid overdoses. We attempted to validate our model by examining the ratio of nonfatal overdoses to fatal overdoses estimated in our model to similar estimates in the literature – Bradley et al (2022)[23] estimated a nonfatal to fatal overdose ratio of 40.8 (95% CI, 20.7–80.6) among people who inject drugs (PWID). Our model identified 25,421 fatal overdoses and 1,002,586 nonfatal overdoses in 2019 under the baseline scenario after calibration, a ratio of approximately 39.43. An important caveat to note is that the estimates in Bradley et al. represent all overdoses among PWID and may not be representative of the ratio among people with OUD.

We note that we do not specifically report the number of nonfatal overdoses in scenarios which model the expected impact of decreases in fatal overdose rates due to concerns around the model's ability to accurately determine any corresponding changes in non-fatal overdoses. These concerns arise because the model considers generic interventions that reduce fatal overdoses overall but does not distinguish which intervention and when those interventions are applied – e.g., in interventions where an overdose has occurred but intervened upon through naloxone, each averted fatal overdose increases the number of nonfatal overdoses correspondingly; conversely, in interventions where the overdose has been prevented in the first place through, say, fentanyl test strips, there are no corresponding changes in nonfatal overdoses for every fatal overdose averted. In a similar vein, we do not report fatal overdoses for scenarios in which we model the expected impact of decreases in nonfatal overdoses since the model cannot accurately determine how interventions that decrease nonfatal overdoses affect fatal overdoses.

© 2024 Nataraj N et al. *JAMA Network Open.*

Additional Model Results

eAppendix 12. Multiple Intervention Scenarios

Model results presented in the main text focus on four scenarios of combined interventions, which are summarized here:

- Scenario A: increased MOUD treatment initiation, decreased OUD recurrence among individuals in early-stage (i.e., MOUD ≤ 6 months) and late-stage treatment (i.e., MOUD >6 months).
- Scenario B: decreased fatal overdoses among the OUD population, decreased fatal overdoses among the MOUD and remission populations, and decreased recurrence among MOUD and remission populations.
- Scenario C: decreased nonfatal overdoses among the OUD population, decreased nonfatal overdoses among the MOUD and remission populations, and decreased recurrence among MOUD and remission populations.
- Scenario D: increased MOUD treatment initiation, decreased fatal overdoses among the OUD population, and decreased fatal overdoses among the MOUD and remission populations.

eFigure 2 depicts model estimates for outcomes of interest across the entire model time horizon for each of these four scenarios relative to the baseline scenario. For scenarios A-D, the model simulation that is depicted corresponds to the simulation where parameters were perturbed by the maximum value for each scenario (this corresponds to the top-right grid in the heatmaps depicted in Figures 2-5 of the main manuscript for each respective model outcome). Over the simulation horizon, we see a slowing of growth in fatal overdoses (Scenarios B and D), nonfatal overdoses (Scenario C), OUD without MOUD prevalence (Scenarios A and D), and an acceleration of growth in MOUD prevalence (Scenarios A and D) and remission (Scenarios A and D). The estimates of these model outcomes at the end of 2023 are reported in eTable 10 for the baseline scenario and scenarios A-D. eTable 11 provides a summary of the relative impact of scenarios A-D on each of the model outcomes by ranking the scenarios in order of greatest to smallest associations for each outcome.

These results highlight that there is no single combination of interventions that has the greatest association with all model outcomes, however, Figure 2 shows that some scenarios have a more noticeable relationship between multiple outcomes than others. Scenario D has the greatest association with fatal overdoses, while Scenario C has the greatest association with nonfatal overdoses, and Scenario A has the greatest relationship with the remaining model outcomes (i.e., OUD without MOUD, MOUD Prevalence, Remission, Other-Cause Deaths). It is also interesting to note that Scenarios A and D predicted decreases in deaths from other causes despite no direct modeled effects on the rates of other-cause mortality from any model state. This finding suggests additional benefits from increasing MOUD treatment initiation, the only intervention common to both Scenarios A and D.

© 2024 Nataraj N et al. *JAMA Network Open.*

eTable 9. Summary of Annual Model Outcomes Under the Baseline Scenario

Year End	Fatal overdoses	Nonfatal overdoses	OUD prevalence (No MOUD)	MOUD prevalence
**2018**	-	-	6,937,920*	1,702,080*
**2019**	25,421*	1,002,586†	8,466,942*	1,915,927*
**2020**	34,181*	1,235,201†	10,657,549*	1,400,794*
**2021**	41,489†	1,475,476†	12,630,729†	1,322,105†
**2022**	48,495†	1,699,107†	14,419,373†	1,477,742†
**2023**	55,253†	1,927,706†	16,072,360†	1,677,988†
**2021-2023 Total**	**145,237†**	**5,102,289†**	-	-

*Calibration estimates
†Projected estimates

eTable 10. Model Outcomes for Baseline and Intervention Scenarios When Interventions are set to the Maximum Effect Size Modeled. Outcomes Reported at end of Model Simulation (end of 2023).

Model Outcome	Baseline	Scenario A: Increasing MOUD initiation and decreasing OUD recurrence	Scenario B: Decreasing fatal overdoses and decreasing OUD recurrence	Scenario C: Decreasing nonfatal overdoses and decreasing OUD recurrence	Scenario D: Decreasing fatal overdoses and increasing MOUD initiation
**Fatal Overdoses**	204,839	199,733	154,000	Not reported**	151,622
**Nonfatal Overdoses**	7,340,076	6,996,129	Not reported**	5,545,368	Not reported**
**OUD without MOUD**	16,072,360	12,310,637	15,817,189	15,673,142	12,818,192
**OUD Prevalence***	17,750,348	16,289,219	17,621,178	17,499,240	16,583,781
**MOUD Prevalence†**	1,677,988	3,978,582	1,803,989	1,826,098	3,765,589
**Remission**	4,478,341	5,993,934	4,661,416	4,737,700	5,741,349
**Other-Cause Deaths**	897,060	848,525	894,050	892,157	854,540

*OUD Prevalence includes the following model states: OUD, MOUD ≤1 month, MOUD >1-6 months, MOUD >6-12 months, MOUD >12 months
†MOUD Prevalence includes the following model states: MOUD ≤1 month, MOUD >1-6 months, MOUD >6-12 months, MOUD >12 months
** We do not report fatal and nonfatal overdoses in scenarios specifically examining interventions which respectively modify nonfatal and fatal overdose rates since the model cannot accurately determine how interventions that decrease one rate influence the other rate (See eAppendix 11).

eTable 11. Summary of Relative Impact on Model Outcomes Across all Multiple Intervention Scenarios.

Relative impact across scenarios	Fatal Overdoses	Nonfatal Overdoses	OUD without MOUD	MOUD Prevalence	Remission	Other-Cause Deaths
**1 (Greatest association)**	D	C	A	A	A	A
**2**	A	A	D	D	D	D

© 2024 Nataraj N et al. *JAMA Network Open.*

**3**	-	-	C	C	C	C
**4 (Smallest association)**	-	-	B	B	B	B

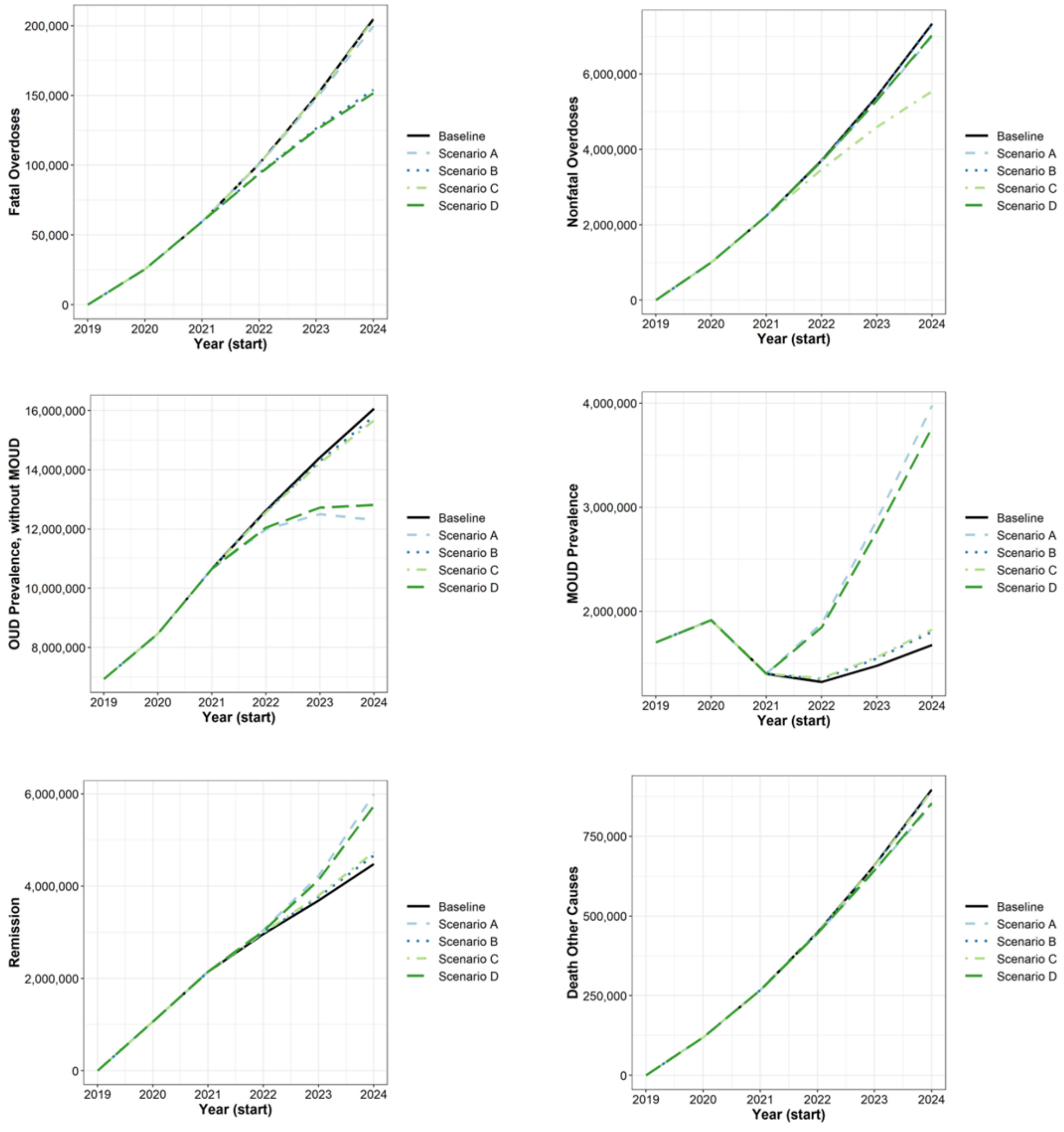

eFigure 2. Comparison of Model Outcomes From Scenarios A to D Over Time Relative to Baseline Scenario.

© 2024 Nataraj N et al. *JAMA Network Open.*

Sensitivity analysis

eAppendix 13. One-Way Parameter Sensitivity Analysis

We performed a one-way sensitivity analysis by varying model parameters individually over a range of given values and report the percent change in model outcomes at the end of the simulation (end of 2023) relative to the baseline scenario. This provides more insight into the dynamics of the simulation model by demonstrating which parameters each model outcome is most sensitive to (i.e., shows the greatest percent change relative to the baseline scenarios) and what range of effect sizes could be expected for each model outcome. Unlike the single intervention and multiple intervention scenarios, the sensitivity analysis does not consider time to implementation, so a change in the parameter assumes the full effect size at the beginning of the 2021 and carries through the end of 2023. Further, we test a wider range of changes in the parameter values (-100% to 200%) that may not be necessarily feasible through public health interventions in the given time frame to understand the behavior of the model in the extremes.

Results of the sensitivity analysis for each model outcome are depicted in eFigures 3-8. These results show that fatal and nonfatal overdoses are most sensitive to the respective rates of fatal and nonfatal overdose from the OUD model state, and secondarily from the remission model state. This most likely reflects the fact that the population in the model is distributed such that there is the highest density of individuals in the OUD state, followed by the remission state. OUD without MOUD, MOUD prevalence, and remission are most sensitive to rates of MOUD initiation, followed by rates of recurrence from MOUD states and rates of nonfatal overdose from MOUD states, though the expected impacts vary across early-stage and late-stage MOUD treatment. Death from other causes is most sensitive to the rate of MOUD initiation, which can likely be attributed to the high other-cause mortality rates among individuals with OUD not receiving MOUD.

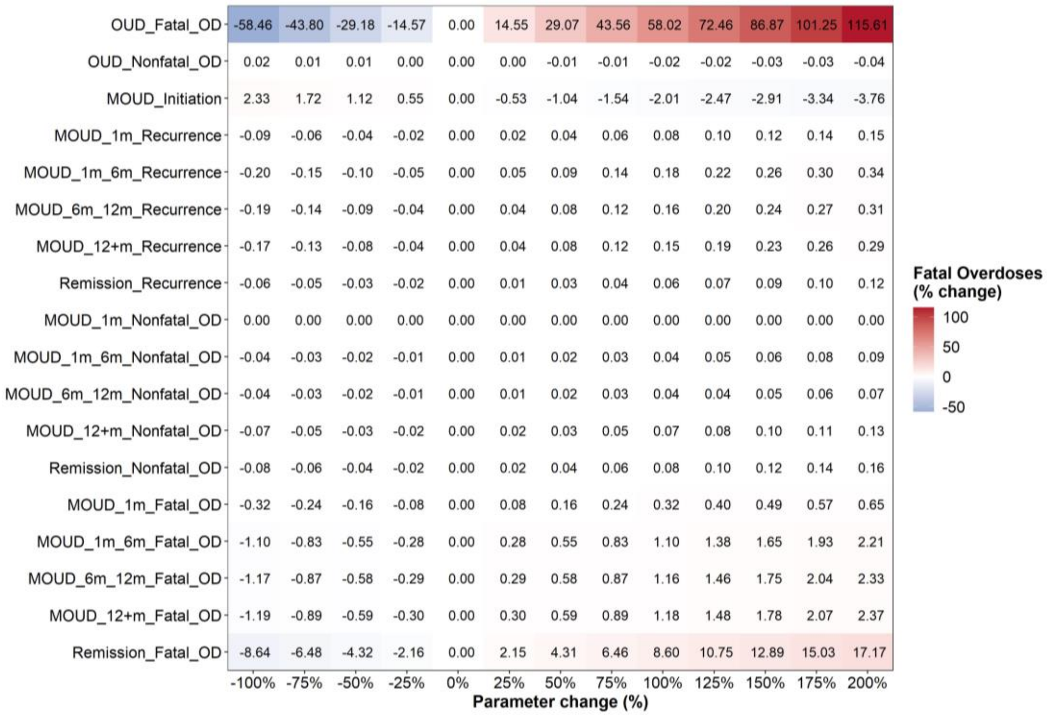

eFigure 3. One-Way Sensitivity Analysis Showing Association Between Parameter Perturbations Ranging From -100% to 200% and Fatal Overdoses Relative to Baseline Scenario.

© 2024 Nataraj N et al. *JAMA Network Open.*

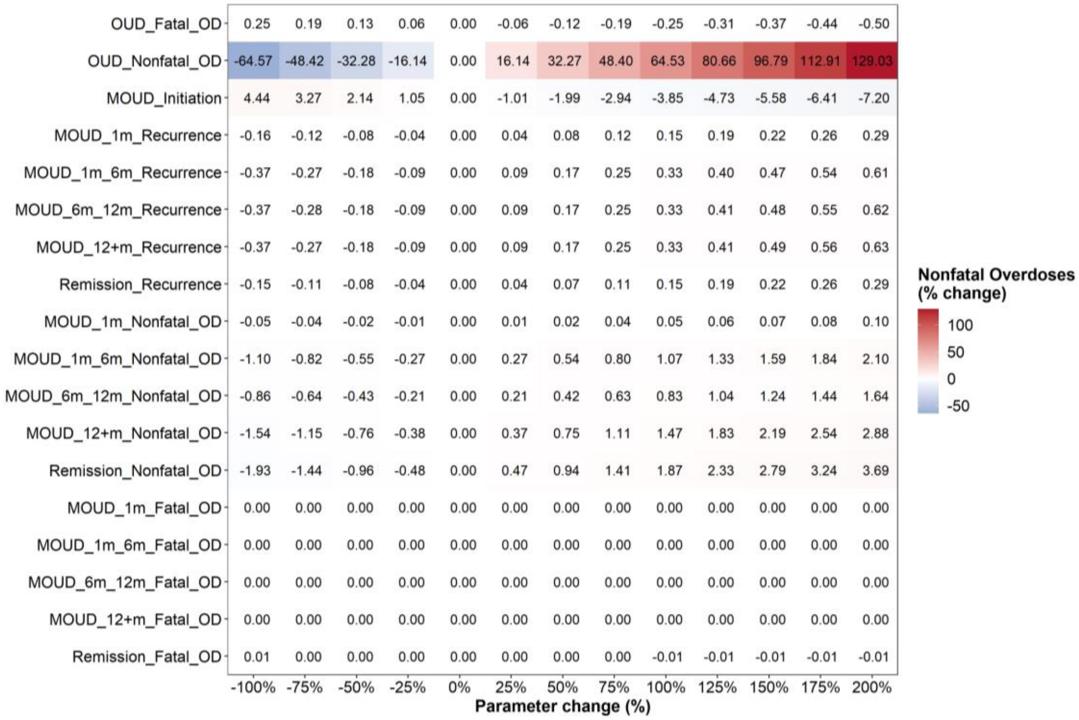

eFigure 4. One-Way Sensitivity Analysis Showing Association Between Parameter Perturbations Ranging From -100% to 200% and Nonfatal Overdoses Relative to Baseline Scenario.

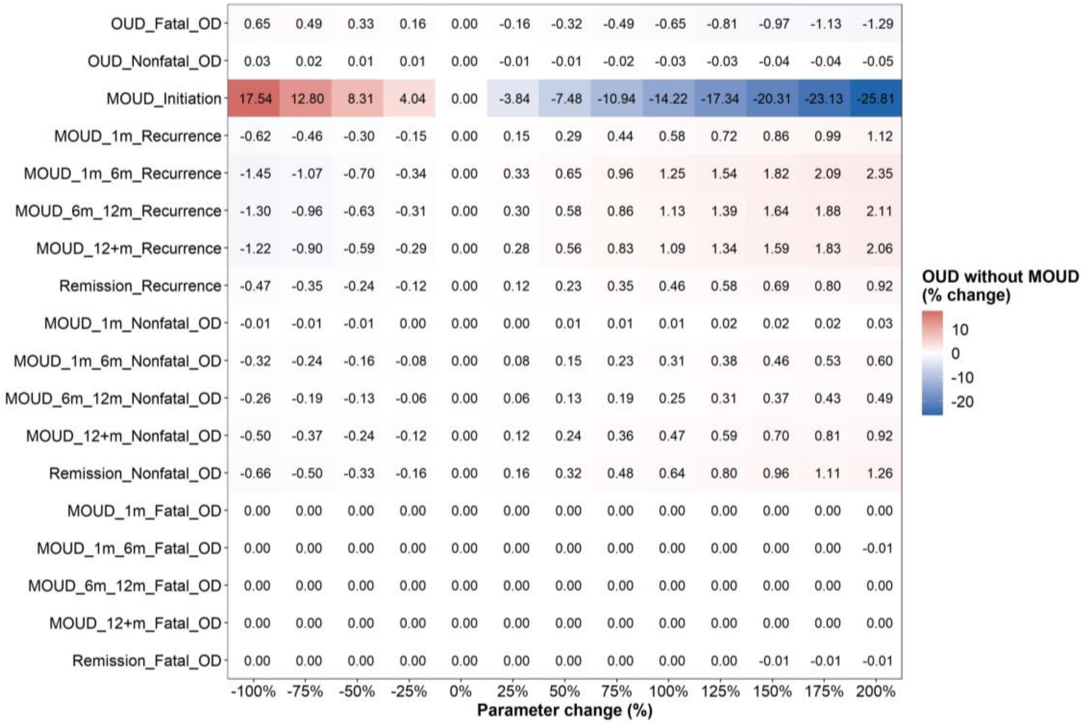

eFigure 5. One-Way Sensitivity Analysis Showing Association Between Parameter Perturbations Ranging From -100% to 200% and Number of Individuals With OUD not Receiving MOUD (OUD Prevalence) Relative to Baseline Scenario.

© 2024 Nataraj N et al. *JAMA Network Open.*

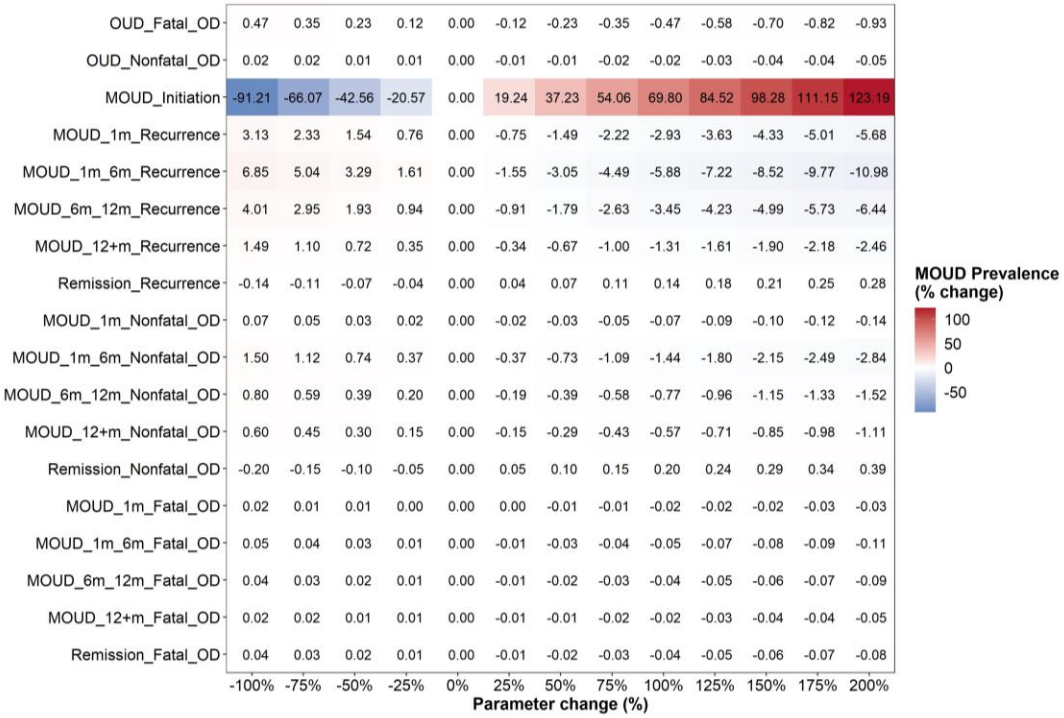

eFigure 6. One-Way Sensitivity Analysis Showing Association Between Parameter Perturbations Ranging From -100% to 200% and Number of Individuals Receiving MOUD (MOUD Prevalence) Relative to Baseline Scenario.

© 2024 Nataraj N et al. *JAMA Network Open.*

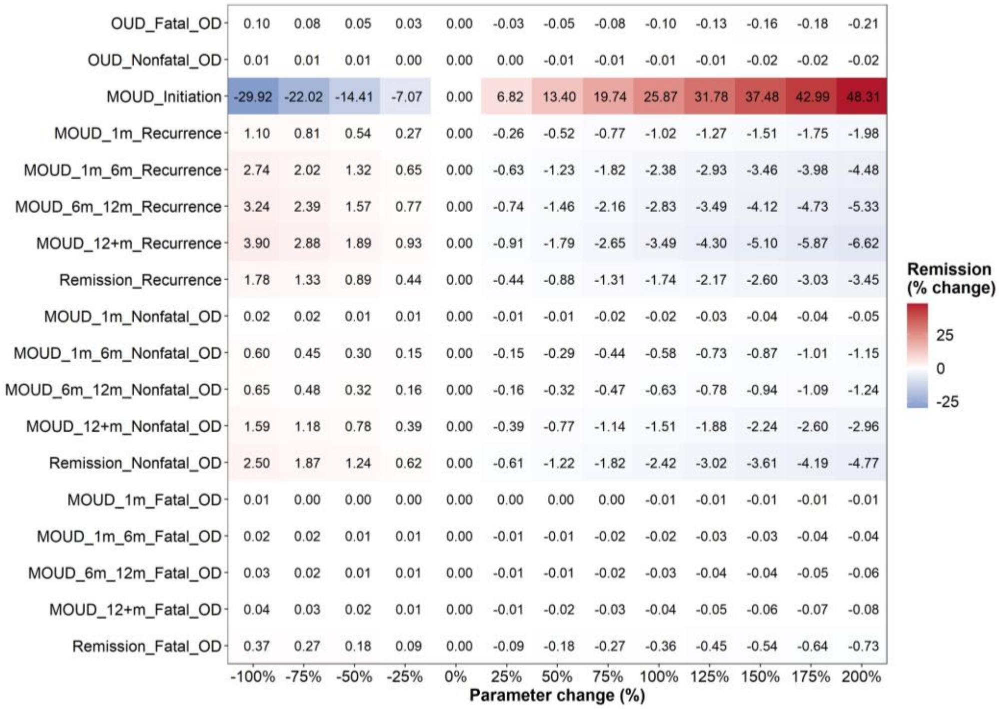

eFigure 7. One-way Sensitivity Analysis Showing Association Between Parameter Perturbations Ranging From -100% to 200% and Number of Individuals in Remission Relative to Baseline Scenario.

© 2024 Nataraj N et al. *JAMA Network Open.*

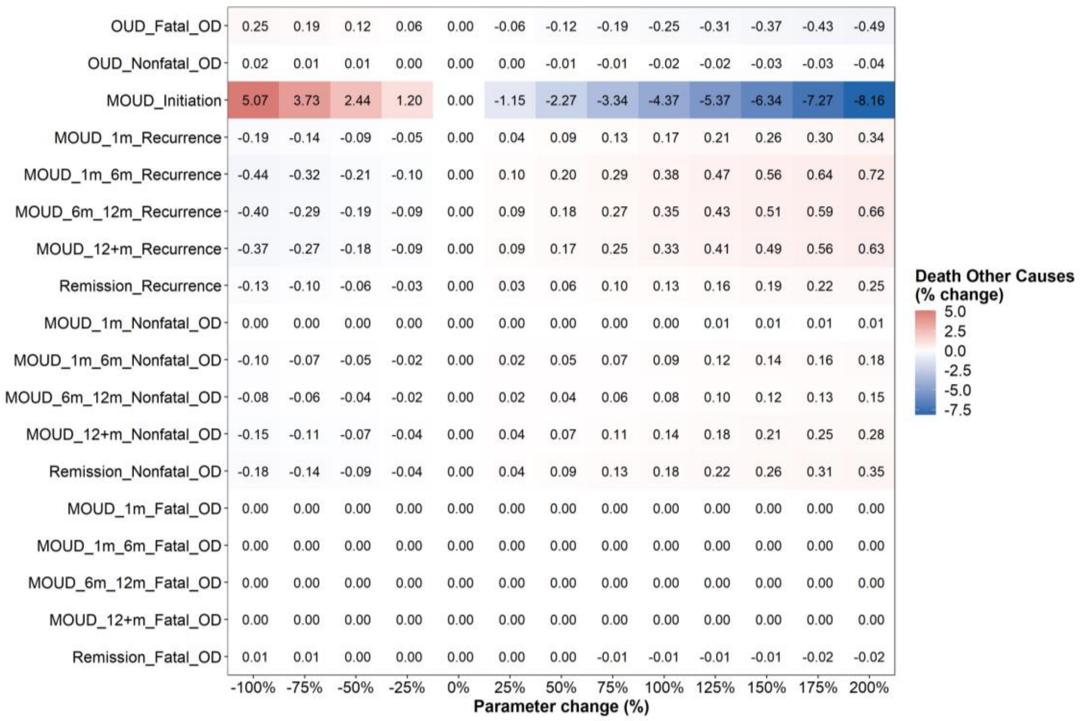

eFigure 8. One-way Sensitivity Analysis Showing Association Between Parameter Perturbations Ranging From -100% to 200% and Deaths From Other Causes Relative to Baseline Scenario.

© 2024 Nataraj N et al. *JAMA Network Open.*

eAppendix 14. Sensitivity Analysis of 2020 OUD Prevalence Data

One-way parameter sensitivity analysis conducted in eAppendix 13 demonstrated that the opioid overdose and OUD prevalence outcomes of interest were most affected by changes in parameters related to inflows and outflows from the OUD model state. As noted previously, this is largely driven by the OUD model state reflecting the highest density of the simulated population relative to other model states. Given concerns around measurement changes that occurred with the 2020 NSDUH survey in part due to the COVID-19 pandemic and the shift from using DSM-IV to DSM-5 criteria to define opioid use disorder[3,24], we conducted additional sensitivity analysis to better characterize the consequences of potentially inaccurate estimates of OUD prevalence as a calibration target on study findings.

For this analysis, rather than use the 2020 adjusted NSDUH estimate of OUD prevalence as reported in eAppendix 8 as a calibration target, we applied the same rate of growth observed between the 2018 and 2019 adjusted NSDUH estimates of OUD prevalence[15] ((10,391,000 - 8,640,000)/8,640,000 = 16.85%) to 2019 as well. By assuming the same growth in OUD prevalence between 2019 and 2020, the new 2020 OUD prevalence for calibration was 12,142,000 (i.e., 10,391,000*1.1685), reflecting a 0.5% increase in OUD prevalence compared to our original estimate obtained in eAppendix 8 (12,076,158).

The results of the baseline scenario under these assumptions are reported in eTable 12, indicating marginally higher numbers of fatal, nonfatal overdoses, and OUD and MOUD prevalence than reported in our main analysis. The relatively low sensitivity of the assumption around the 2020 calibration target was further demonstrated when we examined the association between the interventions implemented in Scenario A (eFigure 9, i.e., increasing MOUD initiation rate and decreasing OUD recurrence rates, and the outcomes of interest. These results were virtually identical to the results presented in the main paper (Manuscript Figure 2). Interventions implemented in Scenarios B, i.e., decreasing fatal overdoses and decreasing OUD recurrence; C, i.e., decreasing nonfatal overdoses and decreasing OUD recurrence; and D, i.e., decreasing fatal overdose rates and increasing MOUD initiation rate (Manuscript Figures 3-5; results not shown) were similarly identical to those presented in the main paper as well.

eTable 12. Summary of Annual Model Outcomes Under the Baseline Scenario Given Sensitivity Analysis of 2020 OUD Prevalence as a Calibration Target.

Year End	Fatal overdoses	Nonfatal overdoses	OUD prevalence (No MOUD)	MOUD prevalence
2018	-	-	6,937,920*	1,702,080*
2019	25,465*	1,004,421†	8,496,594*	1,918,910*
2020	34,233*	1,244,842†	10,786,458*	1,408,751*
2021	42,037†	1,496,657†	12,850,001†	1,337,953†
2022	49,330†	1,730,841†	14,721,359†	1,502,473†
**2023**	56,363†	1,969,594†	16,450,428†	1,711,483†
**2021-2023 Total**	147,731†	5,197,093†		

*Calibration estimates

†Projected estimates

© 2024 Nataraj N et al. *JAMA Network Open.*

Fatal Overdoses

Baseline scenario estimate* = 147,731 fatal overdoses

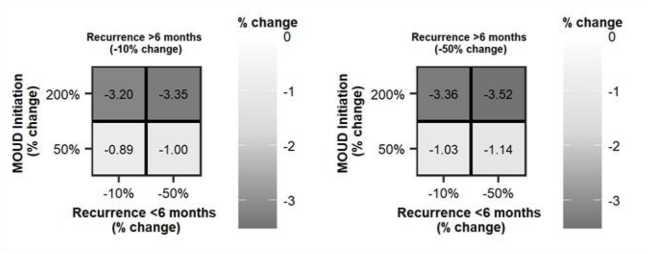

Nonfatal Overdoses

Baseline scenario estimate* = 5,197,093 nonfatal overdoses

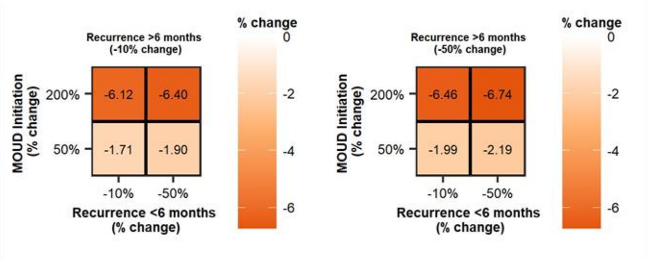

OUD Prevalence without MOUD

Baseline scenario estimate† = 16,450,428 OUD prevalence

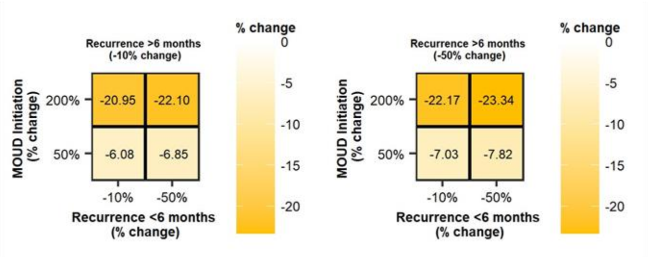

MOUD Prevalence

Baseline scenario estimate† = 1,711,483 MOUD prevalence

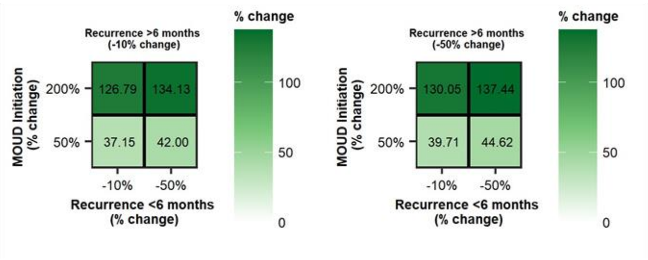

eFigure 9. Percentage Change in Projected Model Outcomes Relative to the Baseline Scenario for Fatal Overdoses, Nonfatal Overdoses, OUD Prevalence (Without MOUD) and MOUD Prevalence at the end of 2023 in model sensitivity analysis reflecting a 0.5% increase in the 2020 OUD calibration target under **Scenario A**: increased treatment initiation (depicted on the y-axis), decreased recurrence during early-stage treatment (MOUD ≤6 months) (depicted on the x-axis), and decreased recurrence during late-stage treatment (MOUD >6 months) (depicted in a series of three plots for each outcome).

Abbreviations: OUD (opioid use disorder); MOUD (medications for opioid use disorder).

*Model estimates are cumulative over the time horizon of simulated public health interventions (January 1, 2021 – December 31, 2023)

†Model estimates indicate prevalence at the end of the simulation (December 31, 2023).

© 2024 Nataraj N et al. *JAMA Network Open.*

eAppendix 15. Sensitivity Analysis of SUDORS Data

To account for possible underestimation of prior OUD in the SUDORS data from limited information present to death investigators as well as known data quality issues during the period of analysis we conducted additional sensitivity analysis on the proportion of decedents with prior OUD. Here, we assumed a 25% increase in the proportion of decedents with reported OUD (0.4973*1.25 = 0.6216), i.e., that 62.16% of decedents with opioid-involved overdoses in SUDORS had a prior OUD. Consequently, using the NVSS 2019-2020 mortality data reported in eTable 7, we assumed the new calibration targets of fatal opioid-involved overdoses in 2019 and 2020 among the population with OUD to be 30,995 (i.e., 0.6216*49,860) and 42,664 (i.e., 0.6216*68,630).

The results of the baseline scenario under these assumptions are reported in eTable 13, indicating marginally higher numbers of fatal, nonfatal overdoses, and MOUD and OUD prevalence than reported in our main analysis. Scenario analysis findings additionally remained highly robust, with percent changes in each of the outcomes under Scenarios A-D remaining identical to results presented in the main paper (Manuscript Figures 2-5; results not shown). This highlights low sensitivity of our main findings to the proportion of decedents with prior OUD.

eTable 13. Summary of Annual Model Outcomes Under the Baseline Scenario Given Sensitivity Analysis of Increases in SUDORS Proportion of Decedents With Opioid-Involved Overdoses and Prior OUD.

Year End	Fatal overdoses	Nonfatal overdoses	OUD prevalence (No MOUD)	MOUD prevalence
2018	-	-	6,937,920*	1,702,080*
2019	31,822*	1,004,307†	8,494,545*	1,918,722*
2020	42,750*	1,240,603†	10,717,792*	1,405,529*
2021	41,742†	1,485,167†	12,728,893†	1,329,626†
2022	48,869†	1,713,209†	14,552,189†	1,488,862†
**2023**	55,743†	1,946,068†	16,237,047†	1,692,772†
**2021-2023 Total**	146,353†	5,144,444†		

*Calibration estimates
†Projected estimates

eReferences

1. Substance Abuse and Mental Health Services Administration. *Key substance use and mental health indicators in the United States: Results from the 2018 National Survey on Drug Use and Health*. Center for Behavioral Health Statistics and Quality, Substance Abuse and Mental Health Services Administration; 2019. (HHS Publication No. PEP19-5068, NSDUH Series H-54). https://www.samhsa.gov/data/report/2018-nsduh-annual-national-report

2. Substance Abuse and Mental Health Services Administration. *Key substance use and mental health indicators in the United States: Results from the 2019 National Survey on Drug Use and Health*. Center for Behavioral Health Statistics and Quality, Substance Abuse and Mental Health Services Administration; 2020. (HHS Publication No. PEP20-07-01-001, NSDUH Series H-55). https://www.samhsa.gov/data/report/2019-nsduh-annual-national-report

3. Substance Abuse and Mental Health Services Administration. *Key substance use and mental health indicators in the United States: Results from the 2020 National Survey on Drug Use and Health*. Center for Behavioral Health Statistics and Quality, Substance Abuse and Mental Health Services Administration; 2021. (HHS Publication No. PEP21-07-01-003, NSDUH Series H-56). https://www.samhsa.gov/data/report/2020-nsduh-annual-national-report

4. Marsden J, Tai B, Ali R, Hu L, Rush AJ, Volkow N. Measurement-based care using DSM-5 for opioid use disorder: can we make opioid medication treatment more effective? *Addiction*. 2019;114(8):1346-1353.

5. Williams AR, Nunes EV, Bisaga A, Levin FR, Olfson M. Development of a cascade of care for responding to the opioid epidemic. *The American journal of drug and alcohol abuse*. 2019;45(1):1-10.

6. Yedinak JL, Goedel WC, Paull K, et al. Defining a recovery-oriented cascade of care for opioid use disorder: a community-driven, statewide cross-sectional assessment. *PLoS medicine*. 2019;16(11):e1002963.

7. Centers for Disease Control and Prevention. Medications for Opioid Use Disorder Study (MOUD Study). Updated 1/24/2022. https://www.cdc.gov/opioids/framework/surveillance-research/moud-study.html

8. Xu KY, Mintz CM, Presnall N, Bierut LJ, Grucza RA. Comparative effectiveness associated with buprenorphine and naltrexone in opioid use disorder and cooccurring polysubstance use. *JAMA network open*. 2022;5(5):e2211363-e2211363.

9. Mojtabai R. Estimating the prevalence of substance use disorders in the US using the benchmark multiplier method. *JAMA psychiatry*. Available online 2022 2022;doi:doi:10.1001/jamapsychiatry.2022.2756

10. Wakeman SE, Larochelle MR, Ameli O, et al. Comparative Effectiveness of Different Treatment Pathways for Opioid Use Disorder. *JAMA Netw Open*. Feb 5 2020;3(2):e1920622. doi:10.1001/jamanetworkopen.2019.20622

11. Sordo L, Barrio G, Bravo MJ, et al. Mortality risk during and after opioid substitution treatment: systematic review and meta-analysis of cohort studies. *BMJ*. Apr 26 2017;357:j1550. doi:10.1136/bmj.j1550

12. Lee JD, Nunes EV, Jr., Novo P, et al. Comparative effectiveness of extended-release naltrexone versus buprenorphine-naloxone for opioid relapse prevention (X:BOT): a multicentre, open-label, randomised controlled trial. *Lancet*. Jan 27 2018;391(10118):309-318. doi:10.1016/S0140-6736(17)32812-X

13. Lee JD, Friedmann PD, Kinlock TW, et al. Extended-Release Naltrexone to Prevent Opioid Relapse in Criminal Justice Offenders. *N Engl J Med*. Mar 31 2016;374(13):1232-42. doi:10.1056/NEJMoa1505409

© 2024 Nataraj N et al. *JAMA Network Open.*

14. Jones CM, Noonan RK, Compton WM. Prevalence and correlates of ever having a substance use problem and substance use recovery status among adults in the United States, 2018. *Drug and alcohol dependence*. 2020;214:108169.
15. Mojtabai R. Estimating the Prevalence of Substance Use Disorders in the US Using the Benchmark Multiplier Method. *JAMA Psychiatry*. Nov 1 2022;79(11):1074-1080. doi:10.1001/jamapsychiatry.2022.2756
16. Goldstein RB, Chou SP, Smith SM, et al. Nosologic Comparisons of DSM-IV and DSM-5 Alcohol and Drug Use Disorders: Results From the National Epidemiologic Survey on Alcohol and Related Conditions-III. *J Stud Alcohol Drugs*. May 2015;76(3):378-88. doi:10.15288/jsad.2015.76.378
17. Hedegaard H MA, Spencer MR, Warner M,. *Drug overdose deaths in the United States, 1999–2020. NCHS Data Brief, no 428.* . 2021.
18. Tanz LJ, Dinwiddie AT, Mattson CL, O'Donnell J, Davis NL. Drug Overdose Deaths Among Persons Aged 10-19 Years - United States, July 2019-December 2021. *MMWR Morb Mortal Wkly Rep*. Dec 16 2022;71(50):1576-1582. doi:10.15585/mmwr.mm7150a2
19. Goldstein RB, Chou SP, Smith SM, et al. Nosologic comparisons of DSM-IV and DSM-5 alcohol and drug use disorders: Results from the National Epidemiologic Survey on Alcohol and Related Conditions–III. *Journal of studies on alcohol and drugs*. 2015;76(3):378-388.
20. Livne O, Shmulewitz D, Stohl M, Mannes Z, Aharonovich E, Hasin D. Agreement between DSM-5 and DSM-IV measures of substance use disorders in a sample of adult substance users. *Drug and alcohol dependence*. 2021;227:108958.
21. Jalali MS, Ewing E, Bannister CB, et al. Data needs in opioid systems modeling: challenges and future directions. *American journal of preventive medicine*. 2021;60(2):e95-e105.
22. Smart R, Kase CA, Meyer A, Stein BD. *Data sources and data-linking strategies to support research to address the opioid crisis*. U.S. Department of Health and Human Services, Assistant Secretary for Planning and Evaluation, Office of Health Policy; 2018. https://aspe.hhs.gov/sites/default/files/private/pdf/259641/OpioidDataLinkage.pdf
23. Bradley H, Hall E, Asher A, et al. Estimated number of people who inject drugs in the United States. *Clinical Infectious Diseases*. Jan 6 2023;76(1):96-102. doi:10.1093/cid/ciac543
24. Center for Behavioral Health Statistics and Quality. *2020 National Survey on Drug Use and Health (NSDUH): Methodological summary and definitions*. 2021. Accessed 12/4/2023. https://www.samhsa.gov/data/
