# Supplementary material for: Public Health Interventions and Overdose-Related Outcomes Among Persons With Opioid Use Disorder
Source: JAMA Netw Open. 2024 Apr 3;7(4):e244617. doi: 10.1001/jamanetworkopen.2024.4617 (PMC10993074; doi:10.1001/jamanetworkopen.2024.4617)
Supplement: Supplement 2. — Data Sharing Statement [file jamanetwopen-e244617-s002.pdf]

Data Sharing Statement

Nataraj. Public Health Interventions and Overdose-Related Outcomes Among Persons With Opioid Use Disorder. *JAMA Netw Open*. Published April 03, 2024.
doi:10.1001/jamanetworkopen.2024.4617

Data
Data available: Yes
Data types: Data (not involving human participants)
How to access data: All data used in the model are made available in the manuscript and accompanying supplementary content.
When available: With publication

Supporting Documents
Document types: None

Additional Information
Who can access the data: Researchers whose proposed use of the data has been approved
Types of analyses: For research purpose
Mechanisms of data availability: All data are made available in the manuscript and supplementary material
